# Evaluation of nutrient characteristics and bacterial community in agricultural soil groups for sustainable land management

**DOI:** 10.1038/s41598-022-09818-1

**Published:** 2022-05-05

**Authors:** Sumeth Wongkiew, Pasicha Chaikaew, Natta Takrattanasaran, Thanachanok Khamkajorn

**Affiliations:** 1 Department of Environmental Science, Faculty of Science, Chulalongkorn University, Bangkok, Thailand; 2 Water Science and Technology for Sustainable Environment Research Group, Chulalongkorn University, Bangkok, 10330 Thailand; 3 Land Development Department, Land Development Regional Office 1, Pathum Thani, 12110 Thailand

**Keywords:** Microbiology, Biogeochemistry, Environmental sciences

## Abstract

The soil bacterial community is critical for understanding biological processes in soils and is used for agricultural soil management. The understanding of microorganisms and ecology in different soil groups classified based on soil properties (e.g., minerals, soil texture, location, nitrogen, phosphorus, organic carbon and pH, among others), is limited. To suggest soil management strategies using bacterial data, we classified soils into four groups based on physical–chemical characteristics and elucidated their relationships with soil nutrient characteristics and the bacterial community in agricultural fields in Saraburi Province, Thailand. Results show that soil groups with high bacterial diversity had positive correlations with total Kjeldahl nitrogen and available phosphorus but were negatively affected by total organic carbon and pH levels. Dominant bacterial genera included *Lactobacillus*, *Phascolarctobacterium*, *Prevotella*, *Clostridium*, *Gaiellales* and *Blautia*. Significant key biomarkers were found (*p* < 0.05). Nutrient-rich soil groups (high available P, acidic pH) were found with genus *Agromyces*, while low nutrient soil groups (low available P, basic pH) were found with *Hydrogenispora*, *Ignavibacterium* and *Bauldia*. Based on co-occurrence networks, organic degrading bacteria functioned with other bacteria at high degrees of interconnections, suggesting organic amendment, biostimulation and biodegradation using nutrient-rich organic substrates could be used for agricultural soil improvements.

## Introduction

Improving soil quality is a vital step to achieving the Sustainable Development Goals (SDGs) of life on land (SDG 15) and other relevant goals of the United Nations^1,2^. Soil health indicators have recently been proposed beyond soil organic carbon stock and soil quality indices. Soil biodiversity, including nutrients and microbiome parameters, has critical functions in determining the overall soil health of a given soil. Such parameters will soon be used as indicators to evaluate soil health and food^3^.

The bacterial community in agricultural soil is critical for maintaining soil health and plant/crop productivity due to the bacterial activities that provide nutrients to soil and plants, such as organic compound degradation, nutrient mineralisation, nitrification and dissimilatory nitrate reduction to ammonium^4,5^. Soil bacteria transform organic residues into plant nutrients, such as amino acids, ammonium, phosphate and potassium, among others^6^. Studies have suggested that maintaining an optimal ecology of agricultural soil bacteria is necessary to promote sustainable agriculture, which reduces environmental footprints from agriculture and increases high nutrient use efficiency^7^. Optimising soil properties (such as nutrients, electrical conductivity, and pH) and agricultural methods (such as maintaining optimum soil organic nutrients, moisture preservation, tillage, irrigation, crop rotation, and fertilization) were reported to improve a healthy soil bacterial community^8,9^. However, a single solution to promote excellent soil quality regarding nutrient availability and the bacterial community for agricultural soil is impractical due to the high complexity of soil characteristics, nutrient compositions, bacterial diversity, climate pattern and soil management practice in each area. Several studies have suggested that management of agricultural soils based on soil characteristics in categorical groups could simplify the management options and applications for agricultural land use, thus making a practical guideline for farmers to improve soil quality with an efficient method for agricultural production^10^.

Globally, several soil classification systems have been developed for different purposes. The USDA-NRCS soil taxonomic system is widely used in many countries. It is a hierarchical soil classification with six levels from general to specific: order, suborder, great group, subgroup, family and series. The taxonomy is classified by the dominant soil formation, the basis of properties, horizons present, typic and arrangement in the soil profile^11^. Thailand has adopted this soil taxonomy and further combines over 300 soil series with similar soil characteristics, properties and crop productivity potentials into 62 groups for non-scientists and farmers to understand the land use suitability rating, soil utility for economic crops and recommendation for soil management^12^. However, the groups of soil series (hereafter soil groups) based on this classification do not reflect soil quality in terms of nutrient transformations and degradation of organic matter, which are associated with bacterial diversity that are key factors for plant production and biogeochemical processes. Soil group classification with supportive bacterial community information could provide details and suggestions for efficient agricultural practices such as manure application, organic farming, biochar amendment, tillage management and improving the soil carbon to nitrogen ratio^13,14^. Moreover, the distinctive bacterial composition abundance of each soil group can be used as a potential long-term bioindicator for soil health. Studies reported that bacterial diversity and beneficial soil bacteria were affected by types of agricultural land use, geographic location and types of soil^13,14^. Therefore, linking soil groups with nutrient characteristics and bacterial communities in each soil group could elucidate more biological insights, which suggest more strategies for agricultural soils management based on soil groups.

Microbiota study based on 16S rRNA gene sequencing is currently widely used for analysis and understanding soil bacterial community because of the efficiency of high-throughput sequencing. This bacterial method allows the powerful analyses of bacterial community composition, ecology and relative abundance, which can be used to link with multi-environmental parameters and soil types^15^. Moreover, bacterial community analysis can be further carried out to understand the connection/symbiosis of bacteria by co-occurrence network analysis and identify key bacterial biomarkers in different groups of soil series, which will help to suggest proper management strategies in sustainable agriculture^8,10^.

Therefore, the overarching target of this study is to suggest the strategies for effective management of agricultural soil using bacterial communities associated with four soil groups from agricultural fields in Saraburi, Thailand. The specific objectives of this study were to (1) evaluate soil nutrient characteristics in different soil groups, (2) determine bacterial diversities and communities and (3) identify the connection of soil properties and key microorganisms based on soil groups. Therefore, the outcome of this study will provide an understanding of the soil bacterial community in association with soil nutrient characteristics and soil groups, suggesting strategies for land use and management based on the ecosystems of the soil microbiome.

## Results and discussion

### Soil groups and nutrient characteristics

**Figure 1:**
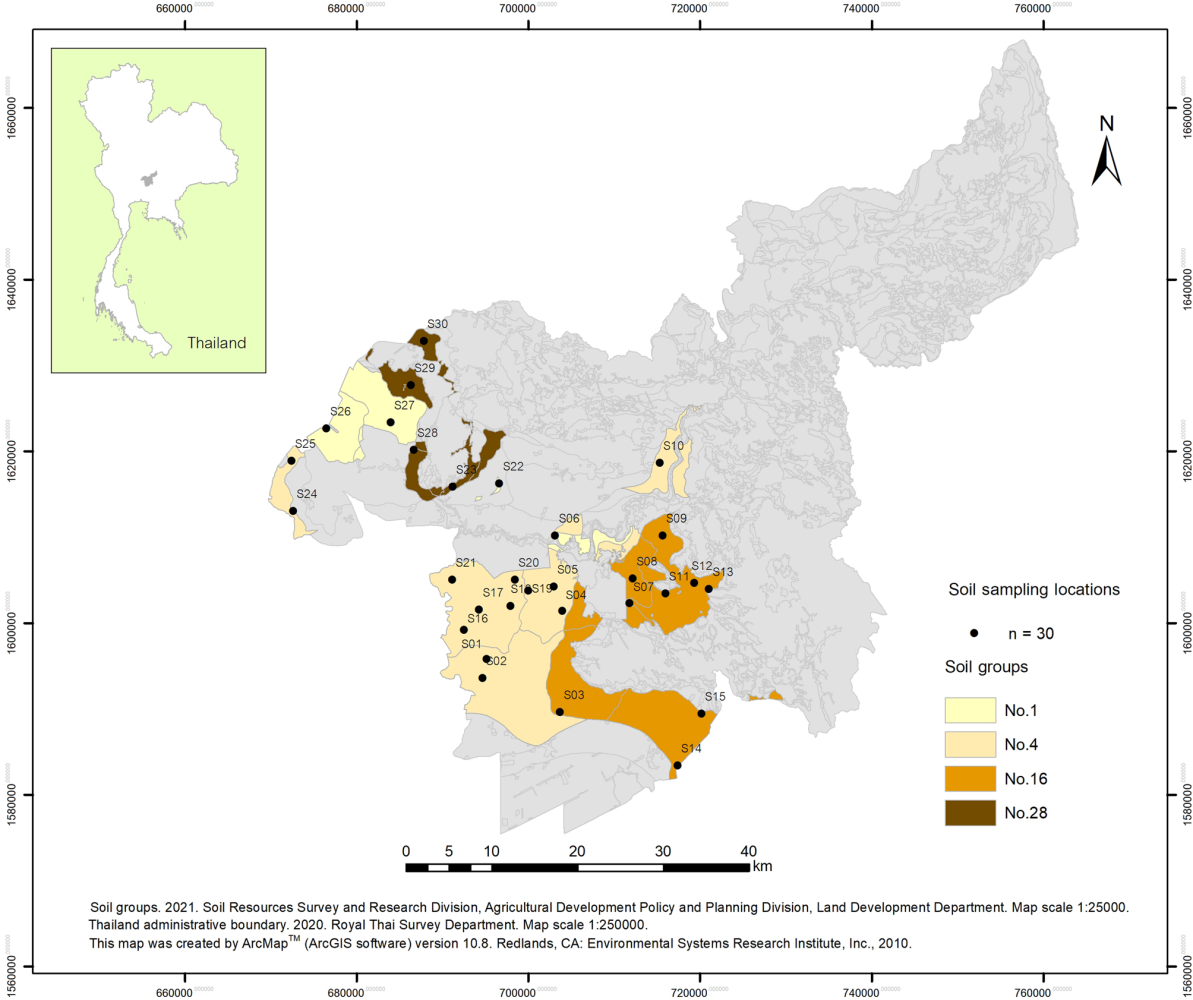
Sampling locations based on soil groups and the study area in Saraburi, Province, Thailand.

**Figure 2:**
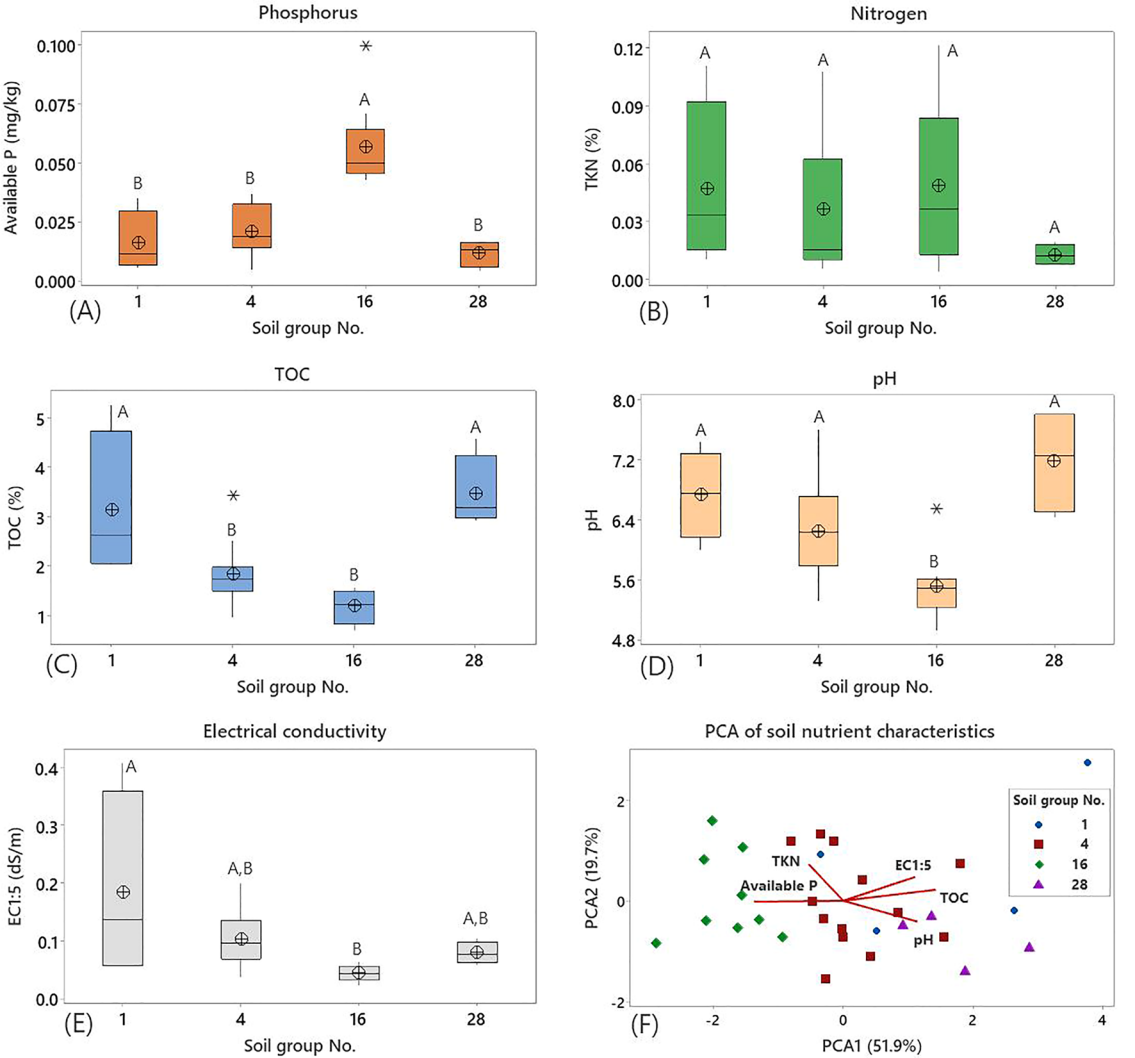
Soil nutrient characteristics including available P (**A**), total Kjeldahl nitrogen (TKN, **B**), total organic carbon (TOC, **C**), pH (**D**), and electrical conductivity (EC, **E**) of different soil groups and principal component analysis (PCA) of the parameters (**F**) in agricultural areas in Saraburi province.

This study categorised soil characteristics based on the guidelines recommended by the Soil Survey and Classification Division, Land Development Department (details in Materials and methods). Soil samples (*n* = 30) were categorised into four groups: No. 1 (*n* = 4), No. 4 (*n* = 13), No. 16 (*n* = 9) and No. 28 (*n* = 4) (Fig. 1). Each soil group showed different physical-chemical characteristics (Fig. 2).

Soil groups No.1, No.4, No.16 and No.28 consisted of different nutrient compositions of available phosphorus (available P), total Kjeldahl nitrogen (TKN), total organic carbon (TOC) and physicochemical properties, namely soil electrical conductivity (EC_1:5_ at 25 °C) and pH levels (Fig. 2 and Table S1 in supplementary data). In this study, TOC contents in soil groups No.1 (3.13 ± 1.51%) and No.28 (3.46 ± 0.76%) were significantly higher compared to groups No.4 and No.16 (*p* < 0.05), which suggested high potentials of soil carbon sequestration in group No.1 and No.28. Soil group No.16 had significantly higher available P contents (0.0567 ± 0.0183 mg/kg) and lower pH levels (5.51 ± 0.45) than other soil groups (*p* < 0.05) (Fig. 2 and Table S1). Lowest average available P (0.01157 ±0.0057 mg/kg) content was found in soil group No.28 (Fig. 2A). Although soil group No.16 shows the highest available P among other soil groups, nutrient contents in the studied site were low in concentrations compared with highly fertilised land with organic management systems^16,17^. Although TKN concentrations did not show significant differences across soil groups (*p* > 0.05), soil group No.16 contained the highest average TKN (0.0486 ± 0.0402%), while the lowest average TKN (0.0127 ± 0.0054%) was found in soil group No.28 (Fig. 2B). TKN negatively correlated with pH levels, and available P negatively correlated with TOC (Fig. 2F). Moreover, the highest average pH level (7.18 ± 0.72) was found in soil group No.28 (Fig. 2D and Table S1). Acidic levels in this range could positively affect the overall nutrient availability of soils (Fig. 2F). The EC_1:5_ values of all soil groups in the topsoil were considered non-saline^18^. This salinity level should be maintained for agricultural purposes in this area, especially for rice cultivation, because the level of slightly saline to strongly saline can lead to lower productivity and quality of rice such as grain yield, growth, and rice aroma^19^.

**Figure 3:**
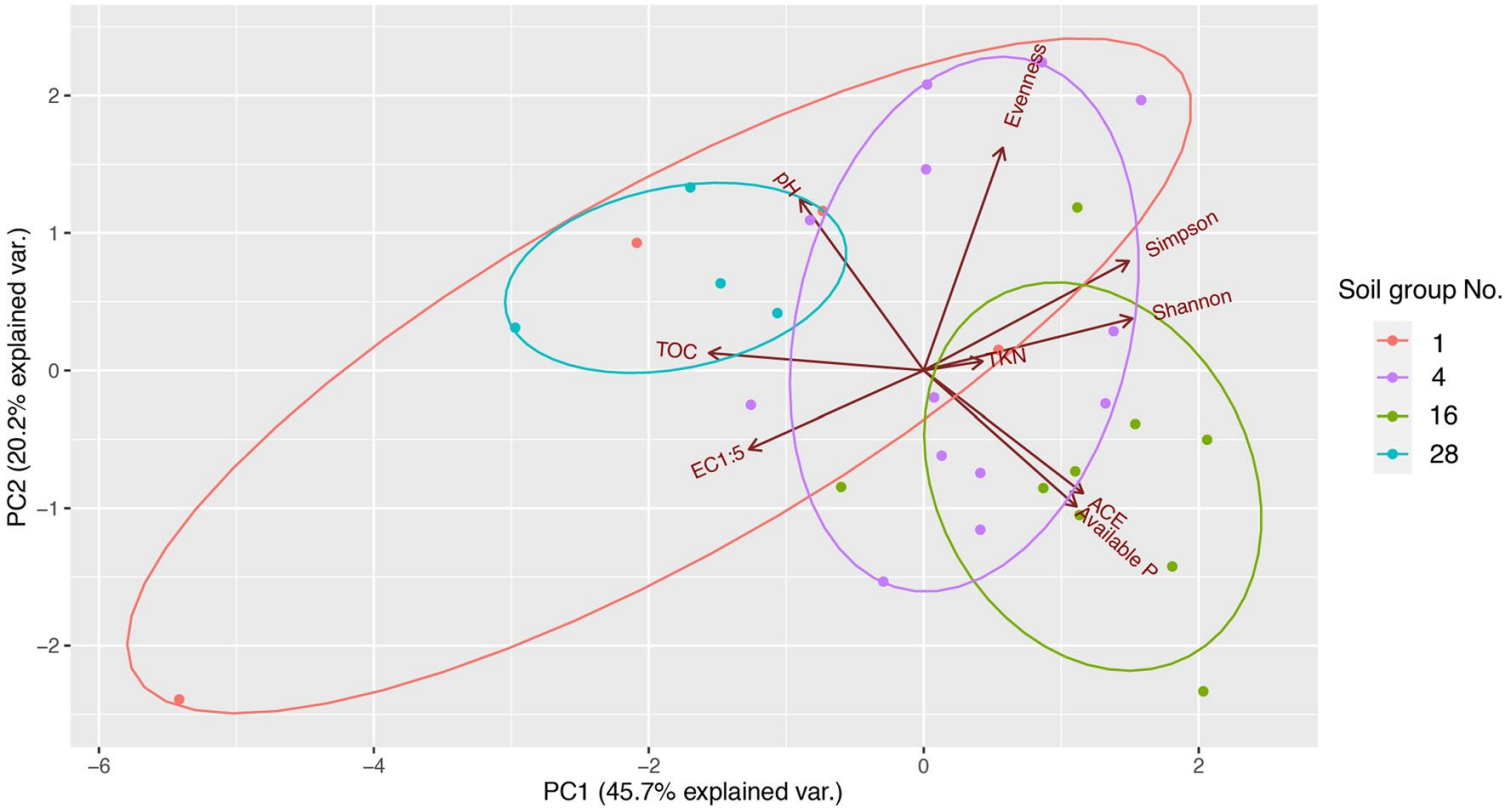
Principal component analysis (PCA) of soil nutrient characteristics and bacterial diversity indices of different soil groups in agricultural areas in Saraburi province. Each soil group No. is represented in red (No.1), purple (No.4), green (No.16), and blue (No.28).

Principal component analysis (PCA) of soil groups and soil nutrient characteristics supported that soil groups classified based on physical characteristics consisted of different nutrient composition characteristics such as TOC, TKN and available P (Fig. 3). For example, samples from the same soil group (No.28) clustered together within their component coordinates with positive correlation with pH and TOC, which were negatively associated with TKN and available P. Soil pH ranging between 4.9 and 7.8 was suitable for mineralisation of organic phosphorus and nitrogen to available P and inorganic nitrogen such as nitrate, while pH levels below 5.5 and above 8.5 limited the available P and inorganic nitrogen for plant uptake^20^. Soil pH influences the bacterial community composition, the diversity and the bacterial abundance, which regulate and promote nutrient availability in the soil^21^. Overall, the findings indicated that soil groups had an effect on varied nutrient availability in soils, implying that soil groups may be used to categorize the TOC, TKN, and available P levels in soil for agricultural land use and management. The differences in soil characteristics could consequently affect soil bacteria, including important bacterial groups for organic decomposition and mineralisation^21,22^.

### Bacterial diversity in different soil types and relationships with soil nutrient characteristics

Over 603 operational taxonomy units (OTUs) were identified as bacterial genera in 30 soil samples with richness ranging from 158 to 240 taxa (Table 1 and Fig. S1). Richness indices (i.e. richness, Chao-1, ACE) among all soil groups were not significantly different (*p* > 0.05). However, the soil bacterial diversity (Shannon and Simpson indices) at the genus level was significantly highest in soil group 4 and lowest in soil group 1 (*p* < 0.05). Bacterial diversities from soil groups No.16 and No.28 are not significantly different among other groups (*p* > 0.05) (Table 1 and Fig. S1). Although soil groups No.16 and No.28 did not show significant differences in bacterial diversity, high bacterial diversity tended to be found in soil group No.4 followed by soil groups No.16, No.28 and No.1, respectively, and the highest richness was more likely to be found in soil group No.16, followed by groups No.4, No.1 and No.28. Bacterial evenness was not found to be significant with soil groups (*p* > 0.05), suggesting the same distribution pattern of bacterial genera in all soil groups. Bacterial 16S rRNA gene sequences obtained from bioinformatic analysis were in a range of 13,480–13,564 reads, which were not significantly different among soil groups (*p* > 0.05). Bacterial diversity indices were negatively affected by TOC, pH and EC_1:5_, but they were positively affected by N and P (Fig. 3). The Shannon and Simpson indices positively correlated with TKN and negatively correlated with TOC and EC_1:5_, indicating that TKN was a key factor that improved bacterial diversity. Abundance-based Coverage Estimator (ACE) of species richness, which was highly correlated with Chao-1 (*R*^2^ = 99.9%, *p* < 0.05) and species richness (*R*^2^ = 99.8%, *p* < 0.05), positively correlated with available P and were negatively affected by high pH level (Fig. 3). Overall, soil group No. 16 was found to have the highest TKN and available P contents and the highest bacterial diversity indices, while soil group No.28 harboured the lowest bacterial diversity indices and the highest TOC content and pH values.

**Table 1:** Bacterial diversity indices and richness of different soil groups.

Soil groups	No. seqs	Richness	Shannon	Simpson	Evenness	Choa-1	ACE
1	13,480 ± 61^a^	168 ± 60^a^	3.96 ± 0.46^b^	0.958 ± 0.028^b^	0.332 ± 0.074^a^	171 ± 61^a^	171 ± 62^a^
4	13,531 ± 54^a^	235 ± 54^a^	4.43 ± 0.25^a^	0.977 ± 0.007^a^	0.380 ± 0.105^a^	240 ± 59^a^	240 ± 58^a^
16	13,564 ± 52^a^	240 ± 68^a^	4.37 ± 0.28^a,b^	0.975 ± 0.008^a,b^	0.349 ± 0.075^a^	247 ± 72^a^	247 ± 72^a^
28	13,502 ± 68^a^	158 ± 13^a^	3.95 ± 0.07^b^	0.963 ± 0.010^a,b^	0.333 ± 0.044^a^	162 ± 14^a^	162 ± 13^a^
Total average	13,530 ± 60	217 ± 63	4.28 ± 0.33	0.972 ± 0.013	0.358 ± 0.086	223 ± 67	222 ± 66

Values reported as mean ± standard deviation. The superscript letters ‘^a^’ and ‘^b^’ represent statistical differences (*p* < 0.05).

Bacterial richness and community are dependent on nutrient characteristics, EC_1:5_ and pH level. The results agreed with other studies, which reported that acidic soil pH negatively correlated with bacterial diversity and likely had a positive correlation with alkaline soil^8,22,23^. Many studies reported that the main driver of soil bacterial diversity was soil pH since the diversity and richness of soil bacterial communities depended on land management and type of vegetation^24^. The results could be because bacteria have different tolerances to soil pH levels, and some bacteria tolerate a narrow pH range^25^. Soil groups and soil conditions (such as nutrient availability, organic carbon, and soil water condition) could promote the abundance of soil bacteria and bacterial diversity^13^. Bacterial diversity and richness were also increased by nitrogen fertilisation^26^. Long-term N-fertiliser caused increases in nitrogen content, resulting in stimulating bacterial growth and their diversity^25^.

### Relationship of bacterial community, soil nutrients characteristics and soil groups

**Figure 4:**
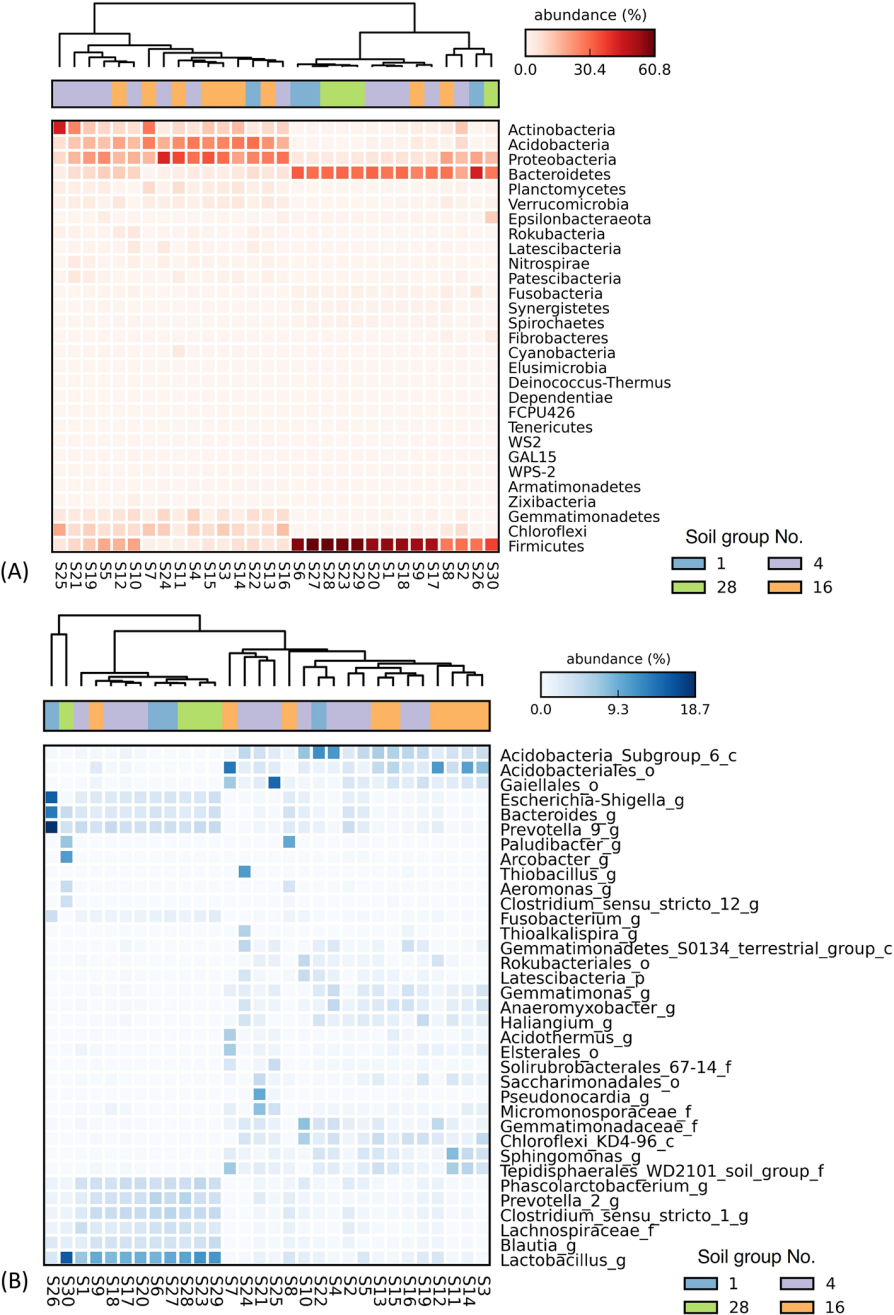
Heat maps of bacterial community compositions at the phylum level (**A**, all phyla) and genus level (**B**, top 35 genera based on relative abundance). Unknown genera are represented appending family (_f), order (_o), class (_c) and phylum (_p) levels while genus is represented by _g. Each soil group No. is represented in blue (No. 1), purple (No. 4), green (No. 28), and orange (No. 16).

**Figure 5:**
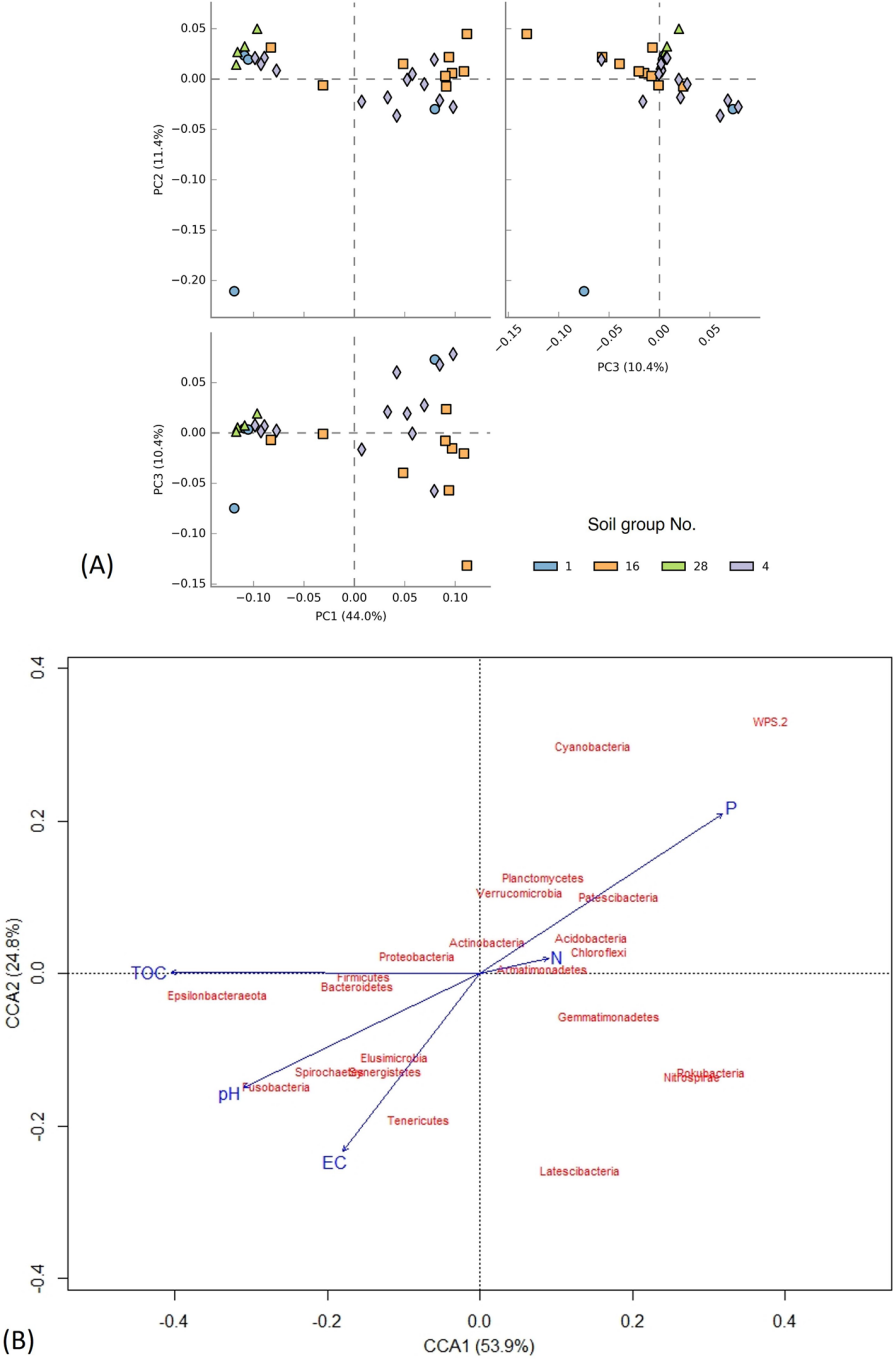
Principal component analysis (PCA) of bacterial phyla from different soil groups (**A**) and canonical correspondence analysis (CCA) of bacterial phyla in response to soil characteristics (**B**). Each soil group No. is represented in blue (No.1), orange (No. 16), green (No.28), and purple (No. 4).

The soil groups harboured several bacterial phyla and genera (Fig. 4), and the bacterial community compositions were not associated with soil groups (Figs. 5A and S2–S5), although each soil group had different nutrient characteristics (Fig. 2). Fig. 4A shows the heatmaps of bacteria at the phylum level, with dominant groups, namely *Firmicutes* (1.5–60.8%), *Proteobacteria* (3.8–42.3%), *Bacteroidetes* (0.3–43.8%), *Acidobacteria* (0.0–29.4%), *Actinobacteria* (0.6–44.4%), *Chloroflexi* (0.0–18.9%), *Gemmatimonadetes* (0.0–10.4%), *Planctomycetes* (0.0–8.4%), *Verrucomicrobia* (0.0–5.1%) and *Fusobacteria* (0.0–4.3%). The *Proteobacteria*, *Bacteroidetes*, *Actinobacteria*, and *Firmicutes* are copiotrophic bacteria (rapid growth at high abundant resource), while *Acidobacteria* is an oligotrophic bacterium (slow growth but adaptable to low nutrients)^17,27^. In this study, *Firmicutes* and *Bacteroidetes* were the dominant phyla, which positively related with soil TOC (Fig. 5B). These phyla also showed a negative relationship with TKN and available P. The next most abundant phylum was *Proteobacteria*, which revealed a positive link with TOC and negative links with TKN and available P. *Proteobacteria* was the main soil-phosphorus-solubilising bacteria, which played an important role in nutrient mineralisation^28^. *Bacteroidetes* had positive correlations with TOC and pH levels. This phylum was found to include facultative bacteria (facultative nutrition) that change their abundances with soil carbon availability^17,29^. The abundance of *Acidobacteria* had a strong positive correlation with available P and TKN but was negatively correlated with TOC and soil pH levels. *Acidobacteria* are oligotrophic bacteria that are extremely sensitive to nutrient availability. *Proteobacteria* and *Bacteroidetes* are primary consumers that could be enriched in high-carbon-availability conditions, while *Acidobacteria* was reported to have high correlations with high phosphorus and nitrogen levels^30^.

The group of dominant bacterial classes (Fig. S6) included *Bacteroidia* (0.3–43.8%), *Clostridia* (0.2–1.3%), *Bacilli* (0.3–15.8%) and *Alphaproteobacteria* (0.0–14.4%), which have been reported with high abundances in high-salinity soil^31^. The group of dominant classes (Fig. S6) also included *Acidobacteriia* (0.0–24.0%), found in medium-salinity soil^31^. *Thermoleophilia* (0.0–22.8%), thermophilic and lipophilic bacteria^32^, were also dominantly found from soils in this study. For bacterial orders (Fig. S7), *Clostridiales* (0.2–41.3%), *Bacteroidales* (0.3–43.7%), *Acidobacteriales* (0.0–18.4%) and *Lactobacillales* (0.1–15.7%) presented as the dominant taxa. The dominant bacterial families (Fig. S8) included *Prevotellceae* (0.2–24.0%), *Lactobacillaceae* (0.1–15.6%), *Lachnospiraceae* (0.0–16.1%), *Ruminococcaceae* (0.1–17.7%), *Gemmatimonadaceae* (0.0–7.9%), *Clostridiaceae* (0.1–5.4%), *Bacteroidaceae* (0.0–13.4%) and *Enterobacteriaceae* (0.0–17.9%). *Ruminococcaceae*, *Lachnospiraceae* and *Clostridiaceae* were found to be dominant with reductive soil disinfestation (biological method for controlling soil-borne disease)^33^ and flooded paddy soil^34^. These families are well known for the degradation and methanogenic decomposition of rice straw and complex materials such as plant materials and cellulose^35^. *Bacteroidaceae* specialise in the degradation of complex organic matter in the biosphere, especially in the form of polysaccharides and proteins^36^.

Top dominant genera include *Lactobacillus*, responsible for carbohydrate and amino acid metabolism (0.1–15.6%)^37^, *Phascolarctobacterium* (chemo-organotroph and obligate anaerobe, 0.0–5.0%)^38^, *Prevotella 2* (0.0–5.5%), *Prevotella 9* (0.1–18.7%, genus found in organic soil samples)^39^, *Clostridium_sensu_stricto_1* (0.0–5.4%, genus associated with manure application in agricultural watershed)^40^, *Gaiellales* (genus found in manure-fertilised soil at neutral pH, 0.0–14.7%)^41^ and *Blautia* (cellulose-degrading anaerobes, 0.0–4.5%)^42^ (Fig. 5B). Most bacterial groups were associated with gut bacteria, suggesting that the beneficial bacteria could be managed and well acclimatised by manure amendment for improving soil quality.

### Bacterial co-occurrence networks in different soil types

**Figure 6:**
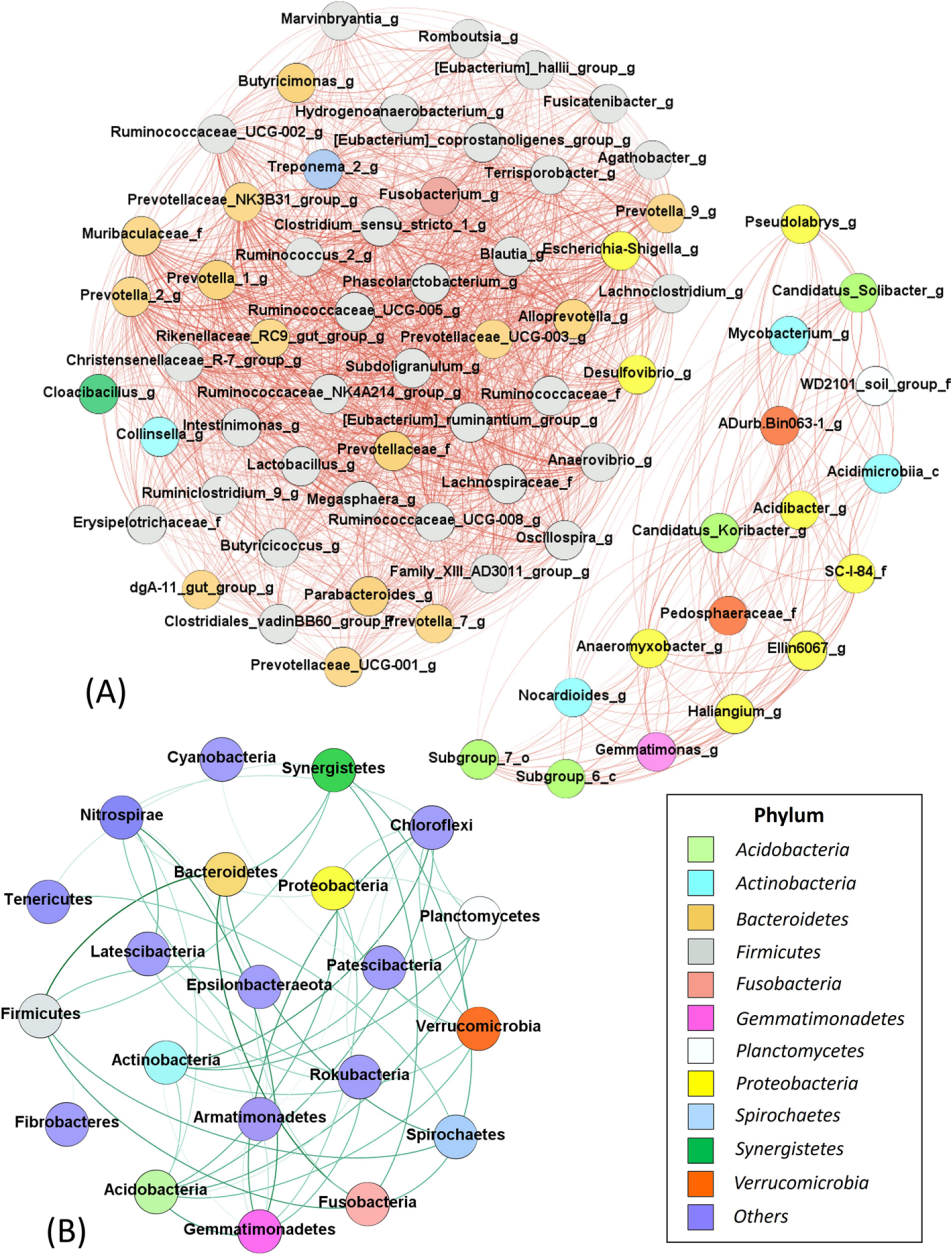
Bacterial community networks at genus (**A**) and phylum (**B**) levels among all samples. Unclassified genera are appended with _f, _o, _c, _p to refer to known family, order, class and phylum, respectively. Co-occurrence nodes and connections were calculated based on Spearman’s correlation using ρ ≥ 0.6 and false discovery rate adjusted *p*-value < 0.01). Thick lines highlight a strong connection. Each bacterial phylum is represented using different colours in the rectangular box.

Keystone bacterial groups were identified using bacterial co-occurrence networks that review the connection of key individual taxa in symbiosis among others (Fig. 6). Co-occurrence analyses at the genus level showed 239 nodes and 6753 edges of all OTUs. Fig. 6A shows genera with a degree of co-occurrence over 75 degrees, 69 nodes and 1423 edges, accounting for 28.9% of total OTUs (average clustering coefficient = 0.98), indicating a strong correlation with low uncertainty of random co-occurrence (see Table S2 for degrees of co-occurrence at genus level). Fig. 6A shows two apparent clusters of OTUs with a stronger connection of multiple genera. Cluster 1 includes *Ruminococus*, *Lactobacillus*, *Prevotella*, *Fusobacterium*, *Blautia*, *Clostridium*, *Treponema*, *Subdoligranulum* and *Phascolarctobacterium* (organic manure associated bacteria)^39,42,43^, which were found at high abundances in soil samples. The other cluster (cluster 2) with lower degrees of co-occurrence included bacterial genera (less abundant than those in cluster 1) such as *Acidibacter* (acidophiles)^44^, *Mycobacterium* (found at acidic soil)^21^, *Koribacter* (associated with nitrogen treatment)^45^, *Pseudolabrys* (associated with nitrogen treatment)^45^, *Anaeromyxobacter* (acidophile)^44^, *Nocardioides* (found in healthy soils)^15^, *Haliangium* and *Gemmatimonas* (organic decomposer and responsible for carbon, nitrogen and phosphorus transformations)^46^. Although these two bacterial clusters were not found to be associated with soil groups, such bacterial genera in cluster 1 were more likely to be found at high abundances in soil groups No.1 and No.28 (Fig. 6A), which consisted of low available P contents, high TOC and high pH levels (Fig. 2). Bacterial genera in cluster 2 were more likely to be found in soil group No.16 (high phosphorus, low TOC and acidic pH level), while soil group No.4 consisted of genera from two clusters. Two main bacterial functions of the soils could be explored based on the two distinct clusters. The results suggest that organic compost amendment with high P contents (struvite and manure) could be used for the soil groups No.1 and No.28 due to the high ability of organic degrading bacteria, while soil group 16 should be managed by reducing chemical fertiliser use to prevent acidic soil using high organic carbon (e.g. straw/ green compost) as the soil harbour mixed functions of bacteria.

At the phylum level, *Firmicutes* (32 nodes) and *Bacteroidetes* (14 nodes) were the top dominant genera in terms of co-occurrence (Fig. 6B). The networks also show the co-occurrence of taxa at phylum levels with 25 nodes and 55 edges (average clustering coefficient = 0.98, Fig. 6B). The results indicate that none of the keystone taxa could have a strong influence on soil groups. Several phyla are interconnected, and the highest degree of co-occurrence was found in *Acidobacteria* and *Chloroflexi* (degree = 9), followed by *Gemmatimonadetes* (degree = 8), *Actinobacteria* (degree = 7), *Nitrospirae* (degree = 7) and *Proteobacteria* (degree = 7) (see degrees of co-occurrence at phylum level in Tables S3). Other bacterial phyla were also important for soil activities as main connectors (Fig. 6B, purple nodes). Overall, the co-occurrence networks of taxa show that bacterial groups from the phylum to the genus levels work as key players in soil environments in all soil samples for organic degradation and nutrient transformation.

### Bacterial biomarkers of different soil types

**Figure 7:**
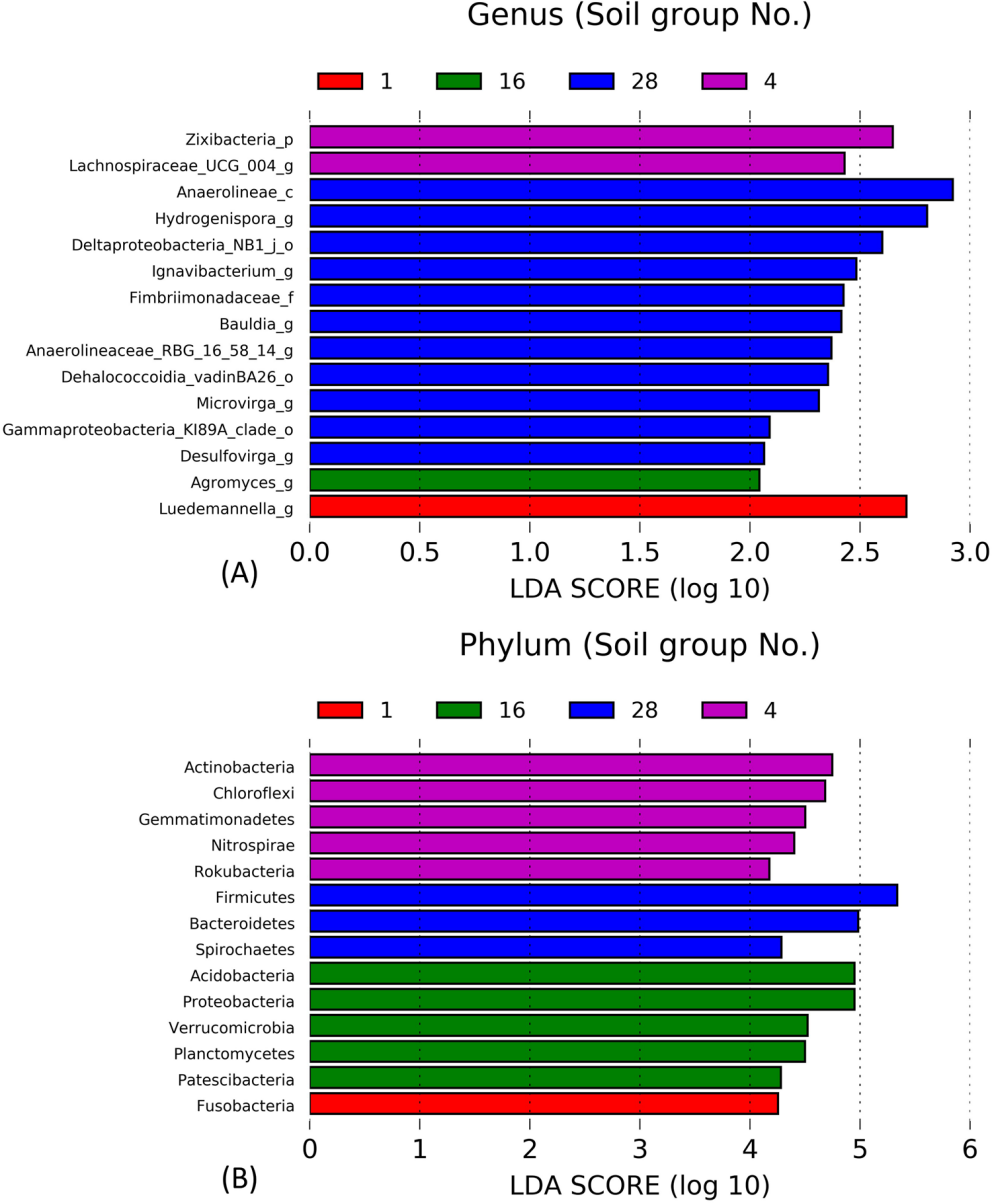
Differential abundance of bacterial communities at the genus (**A**) and phylum levels (**B**) (LDA score > 2) based on linear discriminant analysis (LDA) effect size (LEfSe) from different soil groups (*n* = 30, *p* < 0.05). Each soil group No. is represented in red (No. 1), green (No. 16), blue (No. 28), and purple (No. 4).

Key biomarkers were found in each different soil group, although some bacteria were not highly abundant. Besides the co-occurrence networks, key bacterial groups were found in each soil group based on their significant difference (*p* < 0.05) by linear discriminant analysis (LDA) effect size (LEfSe). There were 1, 4, 1 and 11 key biomarkers at the genus level (including unclassified genera) found at soil groups No.1, No.4, No.16 and No.28, respectively (LDA score > 2, Fig. 7A). At the phylum level, soil group No.1 shows one biomarker, followed by 5, 5 and 3 biomarkers for soil groups No.4, No.16 and No.28 respectively (Fig. 7B). It was found that at soil group No.16 (highest phosphorus level, lowest TOC content), genus *Agromyces* (healthy soil indicator)^15^ and phyla such as *Acidobacteria* (rhizobacteria)^47^, *Proteobacteria* (phosphorus solubiliser)^28^, *Verrucomicrobia* (rhizobacteria) ^47^ and *Planctomycetes* (ammonium oxidiser)^48^ were identified as key biomarkers. Soil group No.28 (highest TOC, poorest soil N and P quality) harboured classified genera such as *Hydrogenispora* (denitrifier)^49^, *Ignavibacterium* (ability in dissimilatory nitrate reduction to ammonium)^49^, *Bauldia* and phyla such as *Firmicutes* and *Bacteroidetes* were key biomarkers. Soil group No.4, which had soil nutrient quality identical to soil group No.1 but lower TOC content and EC_1:5_, showed key biomarkers such as *Lachnospiraceae_UCG_004* (associated with reductive soil disinfestation and pH decrease), unclassified genus of *Zixibacteria* (phylum), and phyla *Actinobacteria*, *Chloroflexi*, *Gemmatimonadetes* and *Nitrospirae*. Soil group No.1 (medium soil quality) had *Luedemannella* (nutrients and energy mediator for bacteria)^50^ and phylum *Fusobacteria* (anaerobe)^24^ as the biomarkers. Soil group No.28 had the highest number of biomarkers, suggesting the bacterial shift was affected by good and poor soil nutrient quality. Therefore, bacterial biomarkers can be used to evaluate soil quality and explore the differences in overall bacterial-driven processes in the agricultural soil groups, which can further suggest strategies for agricultural soil quality improvements for different soil groups.

### The perspective of agricultural land management based on soil group and bacterial keystone biomarkers

Soil group No.1 (high TOC, medium soil nutrient quality) had *Luedemannella* and *Fusobacteria* as biomarkers. These biomarkers suggest that nutrient transformation occurred under anaerobic conditions due to the poorly drained clay texture of the soil group. Organic molecules might be degraded/fermented anaerobically by the bacterial activities in this soil group. At this condition, denitrification could contribute to nitrogen loss in the forms of dinitrogen and nitrous oxide, which is a potent greenhouse gas. To manage this soil group, it is suggested to reduce the water flooded zone and use tillage (for soil respiration) to prevent denitrification and methanogenesis, which cause nitrogen loss and greenhouse gas emissions from nitrous oxide and methane. Anoxic and anaerobic conditions should be avoided.

Soil group No.4 (medium soil nutrient quality) had *Lachnospiraceae_UCG_004*, *Actinobacteria* (copiotrophic organic decomposer) and carbon, nitrogen and phosphorus transforming bacteria such as *Actinobacteria*, *Chloroflexi*, *Gemmatimonadetes* and *Nitrospira*e as biomarkers. These bacterial groups also indicated mixed bacterial functions in soil that facilitate plant growth. This soil group contained better beneficial biomarkers than soil group No. 1. Soil group No. 4 can be managed by organic amendment such as manure/compost that contains high phosphorus concentration with biochar that could capture and store phosphorus in the soil. Degradation of the organic amendments by the bacterial biomarkers of soil group No. 4 could facilitate phosphorus mineralisation from the phosphorus-rich organic compounds contained in that amendments. Thus, organic management with high P is recommended.

Soil group No. 16 (highest available P, acidic soil) harboured *Agromyces*, *Acidobacteria*, *Proteobacteria*, *Verrucomicrobia* and *Planctomycetes* as key biomarkers. The soil groups contained beneficial bacteria, including oligotrophic and copiotrophic bacteria and contained phosphorus solubiliser bacteria, ammonia oxidiser bacteria and rhizobacteria, which indicate good soil health. However, this soil group had an acidic pH level. Thus, to manage this soil group, alkalinity is especially recommended to add to the soil. A high load of ammonia nitrogen should be avoided to prevent acidification of the soil from nitrification. Soil quality improvement may require the application of high alkalinity organic compounds such as manure and organic compost. Acidification from high fertilisation must be watched for.

Soil group No.28 (poor nitrogen and phosphorus, high TOC and slightly alkaline soil) contained biomarkers of the genera *Hydrogenispora*, *Ignavibacterium* and *Bauldia* as well as the phyla *Firmicutes* and *Bacteroidetes*. These bacterial groups indicate denitrification and biodegradation of organic carbon by heterotrophic and copiotrophic bacteria. The slight alkaline condition could be caused by denitrification under anoxic conditions and high organic carbon in this soil group. However, because this soil group contained low nitrogen and phosphorus, it is specifically recommended that fertilisation of this soil group must be the primary concern. Fertilisation using manure or high-nutrient organic compounds such as organic fertilisers is needed for this soil group. Nutrient leaching and dissipation to soil and groundwater should be reduced by using organic matter as soil cover, such as leaves and straw residual materials.

Overall, the results suggest that healthy bacterial communities in soils could be maintained by adding organic matter at near-neutral pH levels. The characteristics of agricultural soils in Saraburi were acidic with bacterial diversity in the range of agricultural soil, which could be affected by the long-term nitrogen chemical fertilisation that is used to accelerate the nutrient levels in agricultural soils and which decreased soil pH levels. Bacterial activities were reported to help in decomposing organic matter and releasing inorganic nutrients for plants by mineralisation. To promote bacterial diversity and beneficial bacteria, proper tillage frequency and improving soil nutrients and carbon storage are also recommended.

## Methods

### Study area

Agricultural areas in Saraburi province, located in central Thailand, were selected as the study sites because the area is comprised of appropriate soil diversity and currently used for commercial agriculture, mostly paddy cultivation (Fig. 1). The eastern part of the province was covered by high plains and plateaus, while the western part was mostly low flat plains, where agricultural activities were mostly utilised for rice cultivation and other non-cyclical crop rotations such as corn and sugarcane plantation. Saraburi has a tropical savanna climate. The climate was arid with little rain in winter, relatively high temperature in summer, cool in winter and rain from May to October. The average annual temperature was 28–29 °C. The study area consisted of four soil series groups as classified by Soil Survey and Classification Division, LDD, including soil group No. 1 (samples S06, S22, S26 and S27, *n* = 4), soil group No.4 (samples S01 and S02, S04 and S05, S10, S16–S21, S24 and S25, *n* = 13), soil group No. 16 (S03, S07–S09, S11–S15, *n*= 9) and soil group No. 28 (S23, S28–S30, *n*= 4).

Soil group No.1 includes poorly drained fine-heavy textured and dark-coloured soils. These soils occupy the low-lying terrain mostly in karst topography and basaltic terrain. In general, they have high fertility status. Soil reaction is neutral to moderately alkaline. Heavy clay soil cracks when dry, making it difficult to prepare for cultivation. This group experiences a lack of water in some places and/or waterlogging in the rainy season. Rice was a main crop cultivated in soil group No. 1. However, non-cyclical crop rotations such as corn and sugarcane plantation were also found in this soil group. Vegetable and fruit plantation can possibly be planted but not recommended due to their high cost for soil improvement both mechanical and biological measures as well as time-labour consuming work.

Soil group No.4 includes mostly somewhat poorly drained, fine-textured (silty clay loam to silty clay) soils. They have moderate fertility. The soil reaction is neutral to slightly alkaline. In some places, salinity can be found in the sub-soils. This group is found in flood-prone topography. Rice cultivation is recommended, but drainage should be taken into consideration for growing other crops or fruit trees. Similar to soil group No. 1., tillage is difficult due to massive texture and cracking characteristics.

Soil group No. 16 includes poorly drained to somewhat poorly drained, medium-textured soils. The soil reaction is strong to very strong acid. Paddy rice is commonly found in this soil group. Lack of water in the dry season, waterlogging in the rainy season, low fertility and massive structure are the main problems of this soil group.

Soil group No. 28, found in the upland area, includes well-drained, dark-coloured heavy clays. They are moderate in fertility. The soil reaction is neutral to moderately alkaline. Upland and tree crops are recommended for this group. The major issue of this group is their shrink-swell properties. Soils become very hard in the dry season and are sticky in the wet season. This soil group is used to grow field crops such as cane and maize.

### Sampling

The soil samples were taken during a mid-rainfall season in when land uses in the study area were active for agricultural activities, and the diversity of bacteria is high at high moisture content conditions. All soil samples are in aerobic/anoxic conditions during the fieldwork. The total sampling size of 30 samples from 30 different sub-districts of the Saraburi province was selected to represent soil characteristics and bacterial community in the study area. The topsoil samples were collected at a 15-cm depth. Each soil sample was transferred to sterile double-zip-lock bags for chemical analyses. Soil samples were divided into two sets. One set was air-dried and sieved using a diameter size of 2.0 mm for soil physical and chemical analyses. The other set was for bacterial analyses, and it was preserved at – 80 °C. Soil samples were sent to the Omics Centre, Chulalongkorn University for 16S rRNA gene sequencing and bacterial community analyses.

### Physical and chemical analyses of soil

The soil pH level was measured at the soil-to-water ratio of 1:1 using a pH meter (HACH, Loveland, CO, USA) (Eckert & Sims, 2009). Total organic carbon was determined by the dry combustion method using a TOC analyser (multi-N/C 3100, Jena Co., Germany). The EC of a 1:5 soil/water (weight/volume) suspension (EC_1:5_) at 25 °C was analysed by Hach sensION156 portable multiparameter probe^51^. TKN was analysed using the Macro-Kjeldahl method^52^. Available P was extracted from the soils using bicarbonate extractant^53^ and was then analysed using the ascorbic acid method^53^. All analyses were conducted in technical triplicate.

### Bacterial community analyses

Bacterial 16S rRNA genes from soil samples were extracted using IANamp Soil DNAKit (Tiangen Biotech, China). Targeted genes were amplified at the V3-V4 regions using the 341F and 805R primers (Forward: TCGTCGGCAGCGTCAGATGTGTATAAGAGACAGCCTACGGGNGGCWGCAG; Reverse: GTCTCGTGGGCTCGGAGATGTGTATAAGAGACAGGACTACHVGGGTATCTAATCC) and sparQ HiFi PCR master mix (Quantabio, USA). Amplification of the genes were carried out with one cycle for initial denaturation (time = 3 minutes, temperature = 94 °C), 25 cycles for denaturation, annealing and elongation (time = 20, 30 or 30 minutes, temperature = 98, 60 or 72 °C) and one cycle for final extension (time = 5 minutes, temperature = 72 °C). Finally, the PCR products were purified using AMPure XP beads and indexed using Nextera XT index primer (5 µL in 50-µL PCR reaction) with 8–10 PCR cycles. Before gene sequencing by Illumina MiSeq, the final PCR products were cleaned and pooled at 6 pM as the final concentration. Clusters and sequences of 250 bp paired-end 16S rRNA reads were generated by Illumina MiSeq. QIIME 2-2019.10 was used as the bioinformatic tool for quality control of the sequencing products and bacterial community analyses including alignment and taxonomic classification^54^. Briefly, raw sequences were demultiplexed and quality filtered using the q2-demux plugin and deionised using DADA2 (via q2-dada2). SEPP q2-plugin with sepp-refs-gg-13-8.qza reference was used to construct a phylogeny followed by a taxonomic classification using the q2-feature-classifier and scikit-learn naïve Bayes classifier against the Greengenes 13_8 reference at 99% similarity. Sequences regarding chloroplast DNA were removed using the taxa filter table in the q2-taxa plugin. All 16S rRNA sequences were deposited to the Sequence Read Archive at The National Centre for Biotechnology Information under accession no. PRJNA776554.

### Data analysis and visualisation

One-way analysis of variance followed by the Tukey-Kramer post-test was used for identifying the significant difference of mean (significance level = 0.05) by Minitab software version 20.4. Correlations among soil nutrient parameters and soil groups were analysed using PCA using R software version 4.1.1. Soil nutrients and parameters were correlated with the bacterial community using the canonical correspondence analysis (CCA) vegan package in R. Bacterial diversity indices (e.g., Shannon diversity index (H’), Simpson's diversity index and evenness index) were calculated using Past software version 4.07^55^. Heatmaps of bacterial community compositions were visualised using Statistical Analysis of Metagenomic Profiles (STAMP) version 2.1.3^56^. Dissimilarity and PCA analyses of bacterial community compositions were analysed and visualised using STAMP version 2.1.3. Bacterial biomarkers of different soil groups were analysed using LEfSe (significance level = 0.05) and visualised for discriminative features using LefSe tools online from Huttenhower lab Galaxy server version 1.0.0^57^. Co-occurrence network analyses for identifying bacterial clusters and bacterial hubs were analysed using a combination of R software and Python3^58^. The bacterial clusters were visualised using Gephi 0.9.2.

### Ethical approval

The paper has been read and approved by all named authors.

## Supplementary Information

Supplementary Information.
