# Supplementary material for: Evaluation of nutrient characteristics and bacterial community in agricultural soil groups for sustainable land management
Source: Sci Rep. 2022 May 5;12:7368. doi: 10.1038/s41598-022-09818-1 (PMC9072534; doi:10.1038/s41598-022-09818-1)
Supplement: Supplementary file 1 — Supplementary Information. [file 41598_2022_9818_MOESM1_ESM.pdf]

Supplementary Information

Evaluation of nutrient characteristics and bacterial community in agricultural soil groups for sustainable land management

Sumeth Wongkiew[1,2], Pasicha Chaikaew[1,*], Natta Takrattanasaran[3], and Thanachanok Khamkajorn[3]

[1]Department of Environmental Science, Faculty of Science, Chulalongkorn University, Bangkok, Thailand

[2]Water Science and Technology for Sustainable Environment Research Group, Chulalongkorn University, Bangkok 10330, Thailand

[3]Land Development Regional Office 1, Land Development Department, Pathum Thani, Thailand

[*]pasicha.c@chula.ac.th

Summary: There are 17 pages of supplementary data, including 3 tables and 14 figures.

Table S1. Soil nutrient characteristics including TOC, TKN, available P, $EC_{1:5}$, and pH levels of different soil groups in agricultural areas in Saraburi province.

Soil groups	TOC (%)	TKN (%)	Available P (mgP/kg)	$EC_{1:5}$ (dS/m)	pH
**1**	3.13±1.51[a]	0.0469 ± 0.0440[a]	0.0158±0.0131[b]	0.184± 0.166[a]	6.73±0.59[a]
**(n=4)**	(2.03-5.27)	(0.0100-0.1108)	(0.0054-0.0349)	(0.056-0.408)	(5.99-7.43)
**4**	1.83±0.61[b]	0.0363 ± 0.0373[a]	0.0207±0.0102[b]	0.101± 0.047[a,b]	6.24±0.65[a]
**(n=13)**	(0.95-3.43)	(0.0053-0.1076)	(0.0042-0.0368)	(0.037-0.198)	(5.31-7.61)
**16**	1.18±0.33[b]	0.0486 ± 0.0402[a]	0.0567±0.0183[a]	0.044± 0.014[b]	5.51±0.45[b]
**(n=9)**	(0.69-1.56)	(0.0040-0.1213)	(0.0423-0.0998)	(0.023-0.064)	(4.92-6.54)
**28**	3.46±0.76[a]	0.0127 ± 0.0054[a]	0.0116±0.0057[b]	0.078± 0.019[a,b]	7.18±0.72[a]
**(n=4)**	(2.91-4.59)	(0.0073-0.0191)	(0.0041-0.0161)	(0.058-0.104)	(6.43-7.80)
**Total**	2.03±1.08	0.0383 ± 0.0369	0.0296±0.0222	0.092± 0.076	6.21±0.80
	(0.69-5.27)	(0.0040-0.1213)	(0.0041-0.0998)	(0.023-0.408)	(4.92-7.80)

Letters a, b, and c represent significant different. Values following ± represent the standard deviation of each concentration (n = 2).

Table S2. Bacterial genera and degrees of co-occurrence (connection) in network diagram representing bacterial connections at genus level in bioponic systems (taxonomic level: p_ = phylum, c_ = class, o_ = order, f_ = family, g_ = genus). Only genera with degrees of co-occurrence ≥ 75 are shown in this table (69 genera).

Genera	Degree
Ruminococcus_2_g	105
Muribaculaceae_f	104
G138	104
G246	102
Subdoligranulum_g	102
Phascolarctobacterium_g	102
Ruminococcaceae_UCG-002_g	101
Prevotella_1_g	100
G249	100
Blautia_g	99
Ruminococcaceae_UCG-005_g	99
Treponema_2_g	97
Prevotella_2_g	96
Prevotellaceae_UCG-003_g	96
Alloprevotella_g	93
G146	93
Terrisporobacter_g	93
G301	91
Fusobacterium_g	91
G330	90
Intestinimonas_g	89
G344	89
Escherichia-Shigella_g	88
Candidatus_Koribacter_g	87
Prevotellaceae_f	87
Lachnoclostridium_g	87
Anaeromyxobacter_g	86
Nocardioides_g	84
Lactobacillus_g	84
G254	84
Desulfovibrio_g	84
Pedosphaeraceae_f	84
Marvinbryantia_g	83
G300	83

Gemmatimonas_g	83
SC-I-84_f	83
Acidibacter_g	83
Cloacibacillus_g	83
Romboutsia_g	82
Ruminococcaceae_UCG-008_g	82
Ruminococcaceae_f	82
Anaerovibrio_g	82
Candidatus_Solibacter_g	81
Lachnospiraceae_f	81
Oscillospira_g	81
Acidimicrobiia_c	80
Butyricimonas_g	80
ADurb.Bin063-1_g	80
dgA-11_gut_group_g	79
G259	79
Butyricicoccus_g	79
Pseudolabrys_g	79
Ellin6067_g	79
Subgroup_6_c	78
G319	78
Haliangium_g	78
Collinsella_g	77
Prevotellaceae_UCG-001_g	77
Parabacteroides_g	77
Agathobacter_g	77
Fusicatenibacter_g	77
Megasphaera_g	77
Subgroup_7_o	76
Prevotella_7_g	76
Erysipelotrichaceae_f	76
Mycobacterium_g	75
Prevotella_9_g	75
Ruminiclostridium_9_g	75
WD2101_soil_group_f	75

Table S3. Bacterial phyla and degrees of co-occurrence (connection) in network diagram representing bacterial connections at genus level in bioponic systems

Phyla	Degree
Acidobacteria	9
Chloroflexi	9
Gemmatimonadetes	8
Actinobacteria	7
Nitrospirae	7
Proteobacteria	7
Firmicutes	6
Planctomycetes	6
Rokubacteria	6
Verrucomicrobia	6
Bacteroidetes	5
Fusobacteria	5
Patescibacteria	5
Spirochaetes	5
Synergistetes	5
Armatimonadetes	4
Epsilonbacteraeota	3
Latescibacteria	3
Tenericutes	2
Cyanobacteria	1
Fibrobacteres	1

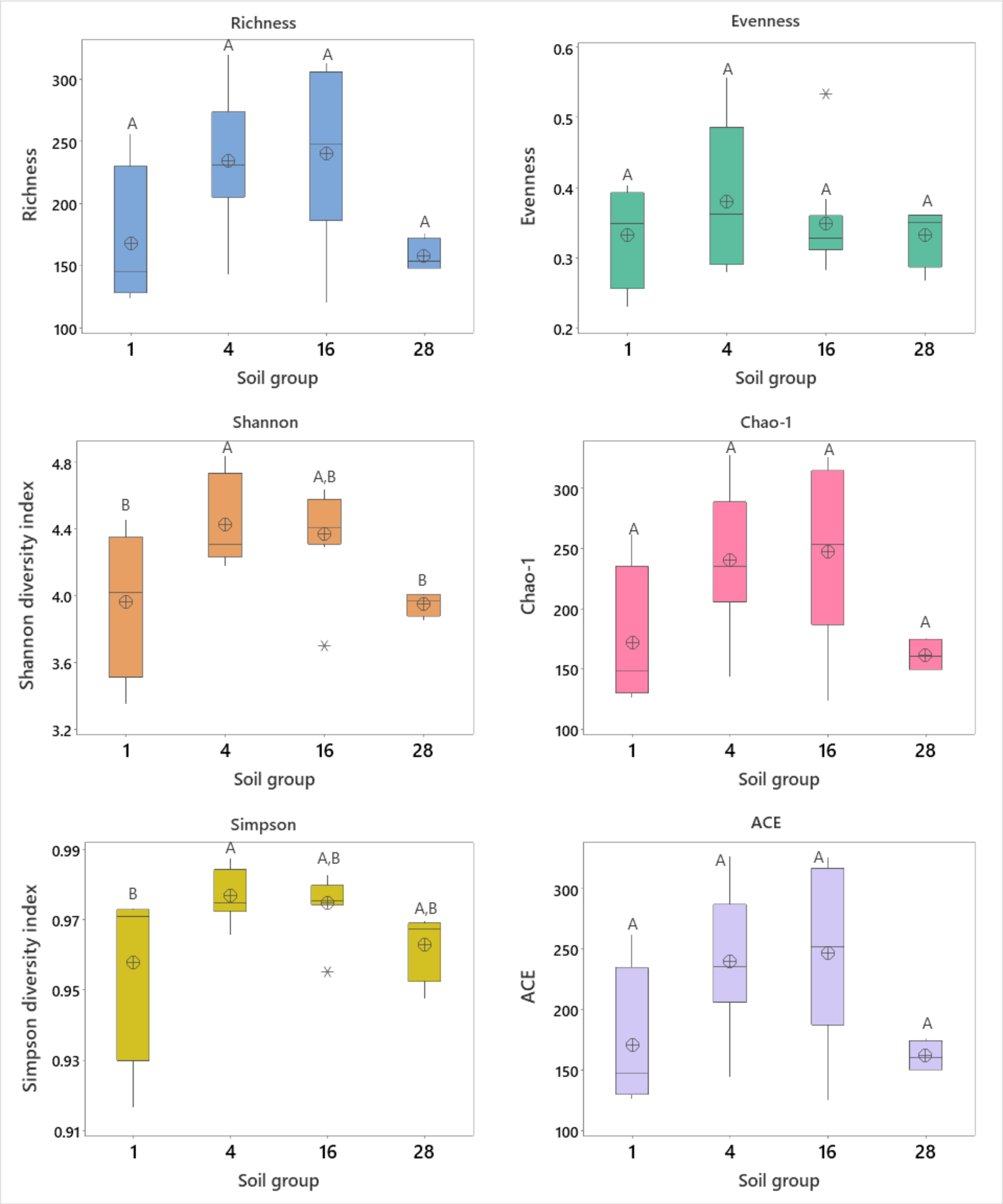

Fig S1. Bacterial diversity indices of different soil groups in agricultural areas in Saraburi province. Letters A and B represent significant different. Error bars represent the standard deviation (n = 2).

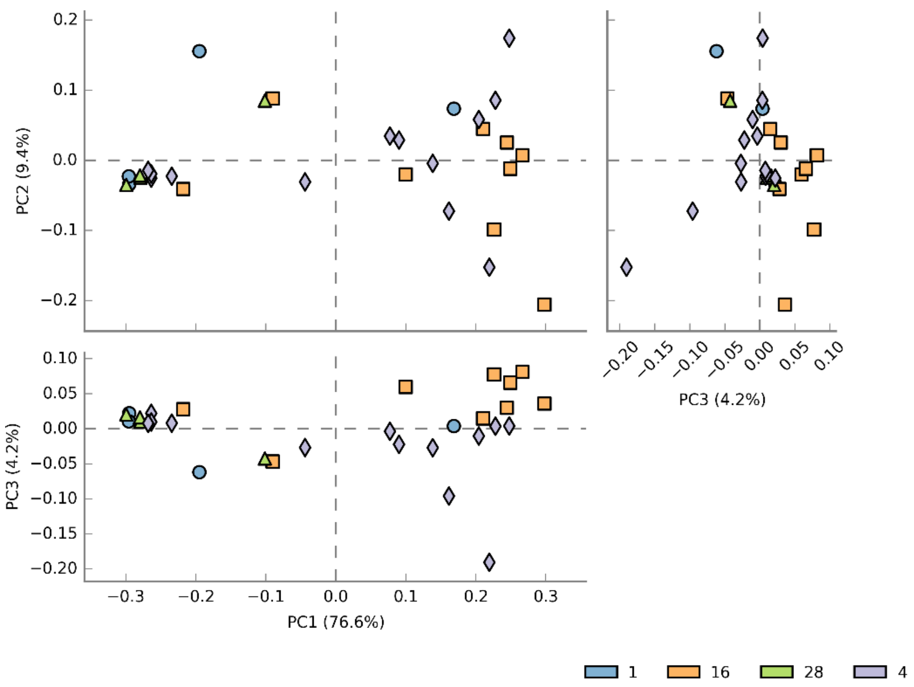

Fig. S2. Principal component analysis (PCA) of bacterial classes from different soil groups.

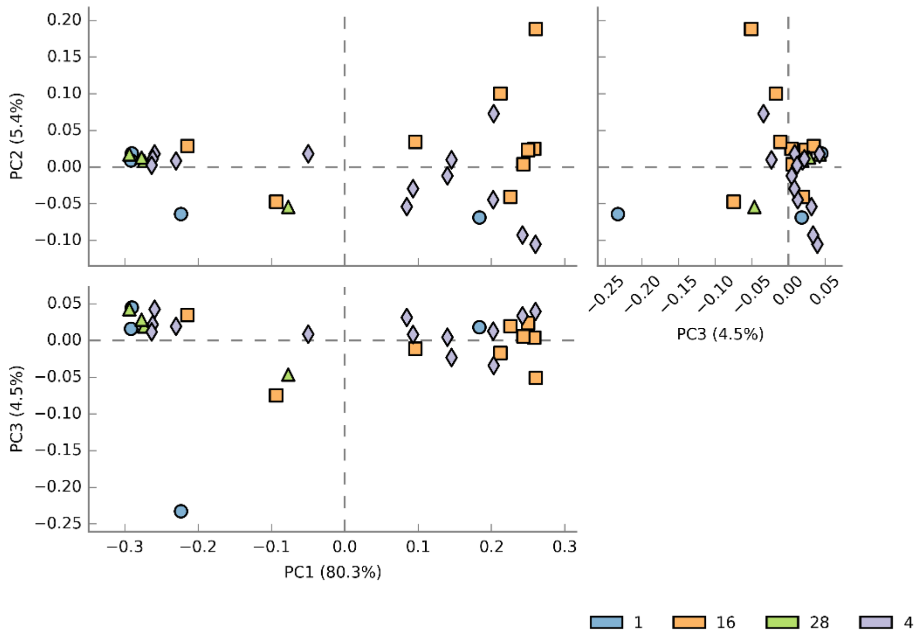

Fig. S3. Principal component analysis (PCA) of bacterial orders from different soil groups.

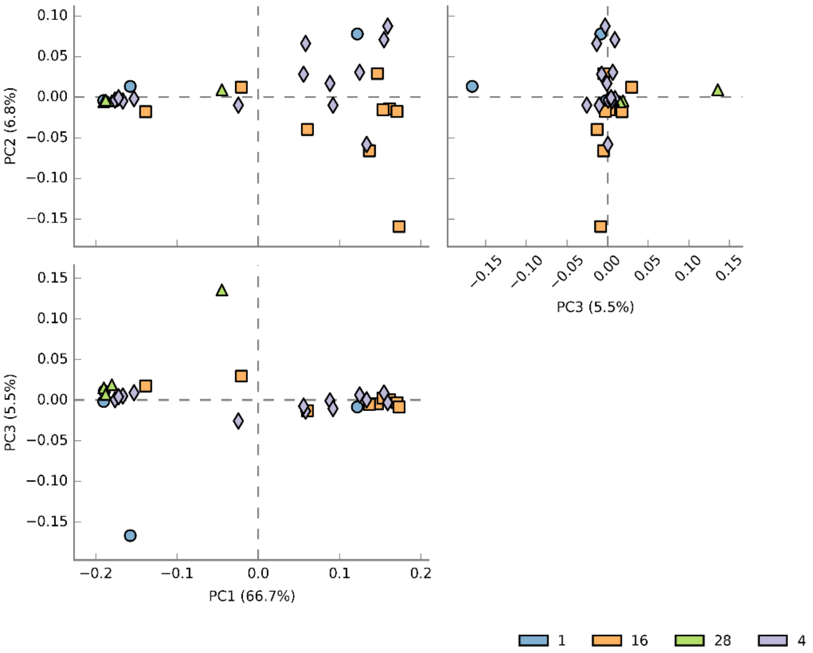

Fig. S4. Principal component analysis (PCA) of bacterial families from different soil groups.

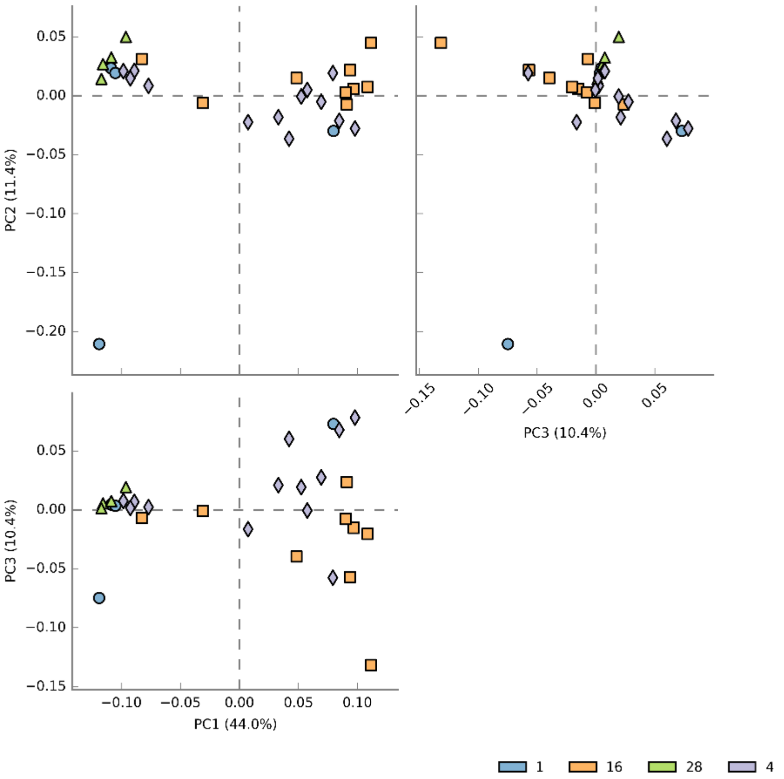

Fig. S5. Principal component analysis (PCA) of bacterial genera from different soil groups.

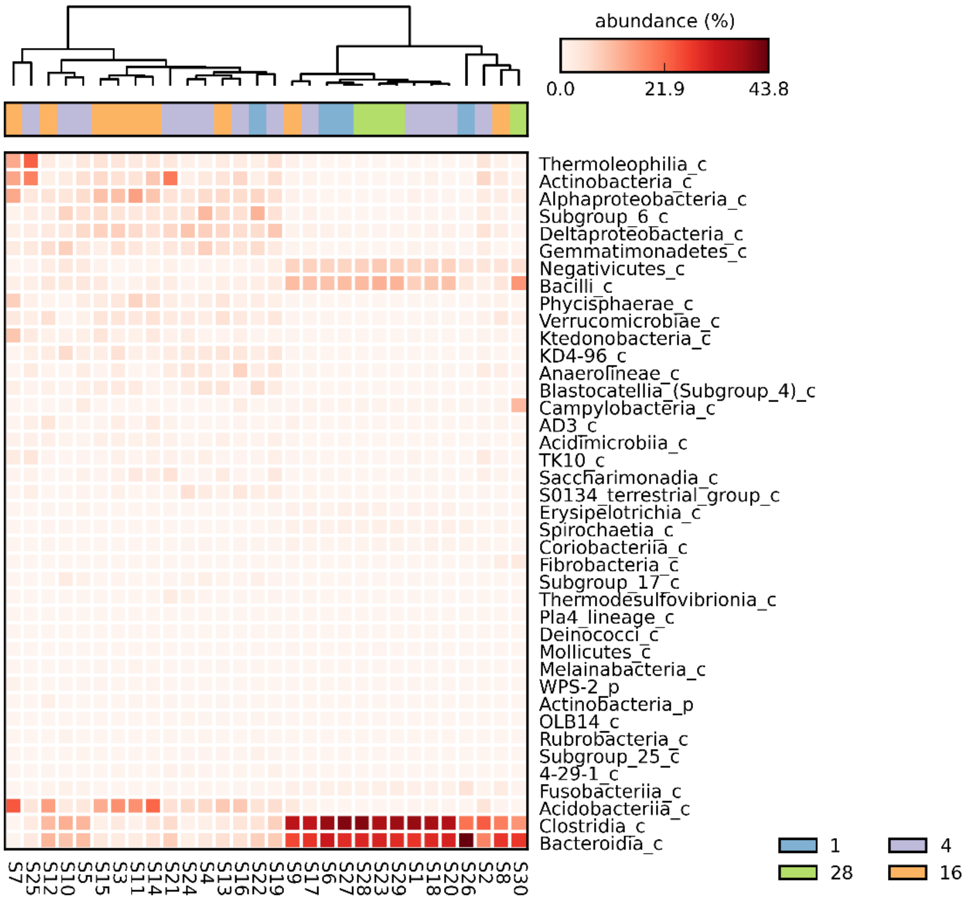

Fig. S6. Heatmaps of bacterial community compositions at class level (top 40). Unknown classes are represented using phylum (_p) levels.

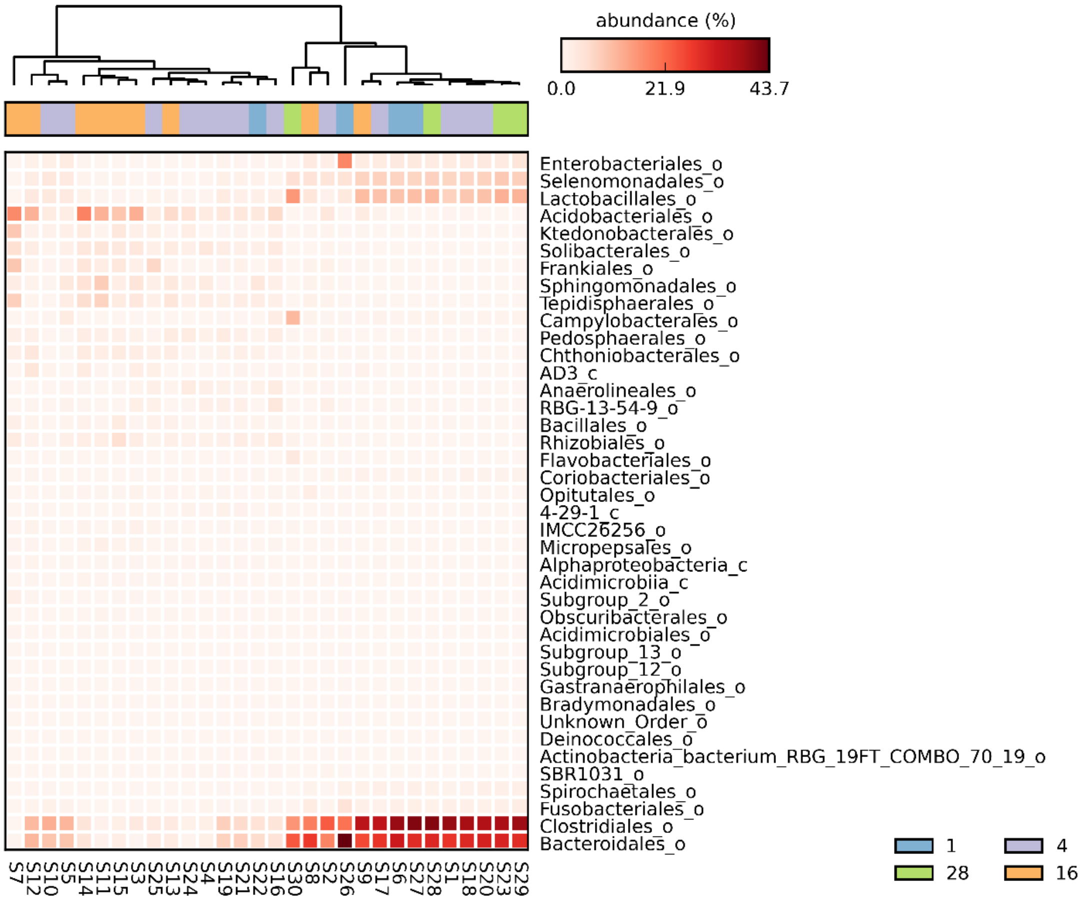

Fig. S7. Heatmaps of bacterial community compositions at order level (top 40). Unknown orders are represented using class (_c) and phylum (_p) levels.

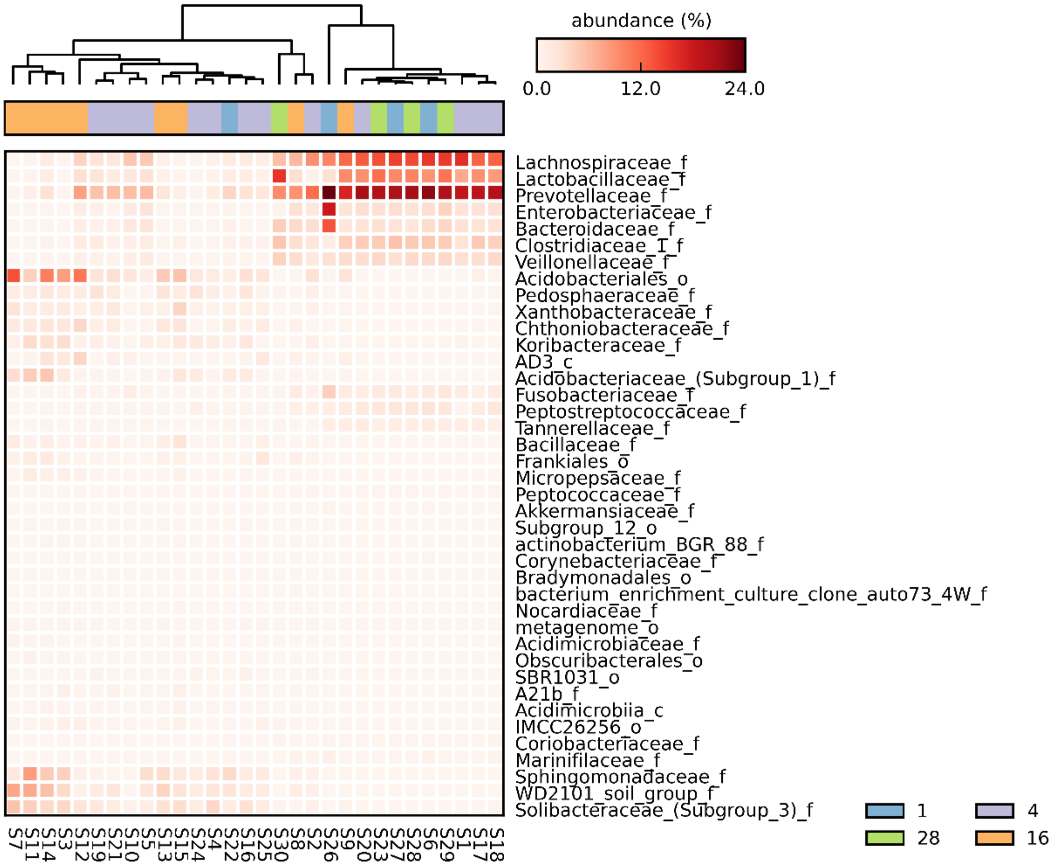

Fig. S8. Heatmaps of bacterial community compositions at family level (top 40). Unknown families are represented using order (_o), class (_c), and phylum (_p) levels.

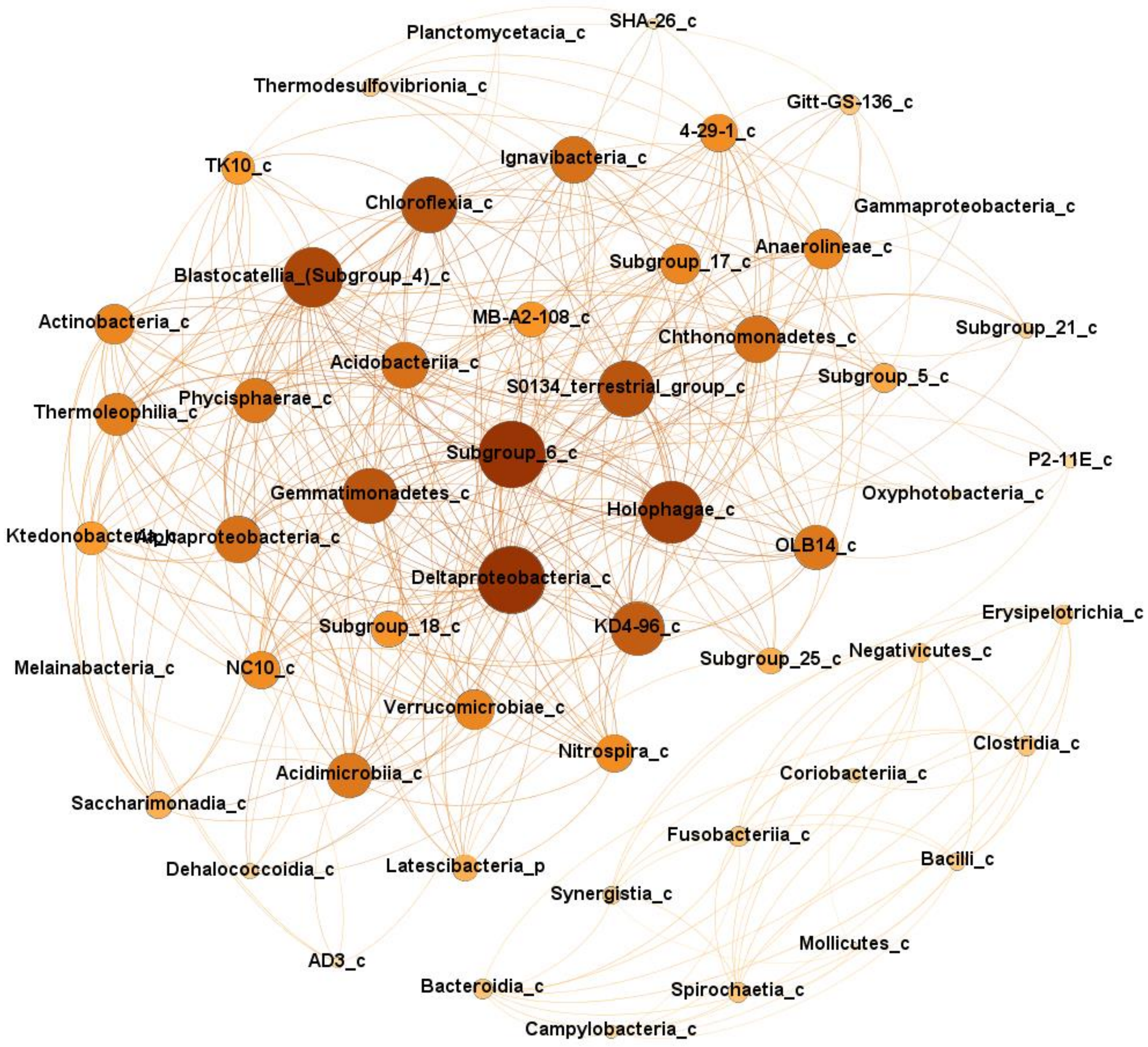

Fig. S9. Bacterial community networks at class level among all samples. Unclassified classes are marked with _p to refer to known phylum. Co-occurrence nodes and connections were calculated based on Spearman's correlation using $\rho \geq 0.6$ and false discovery rate adjusted p-value < 0.01). Thick lines highlight a strong connection.

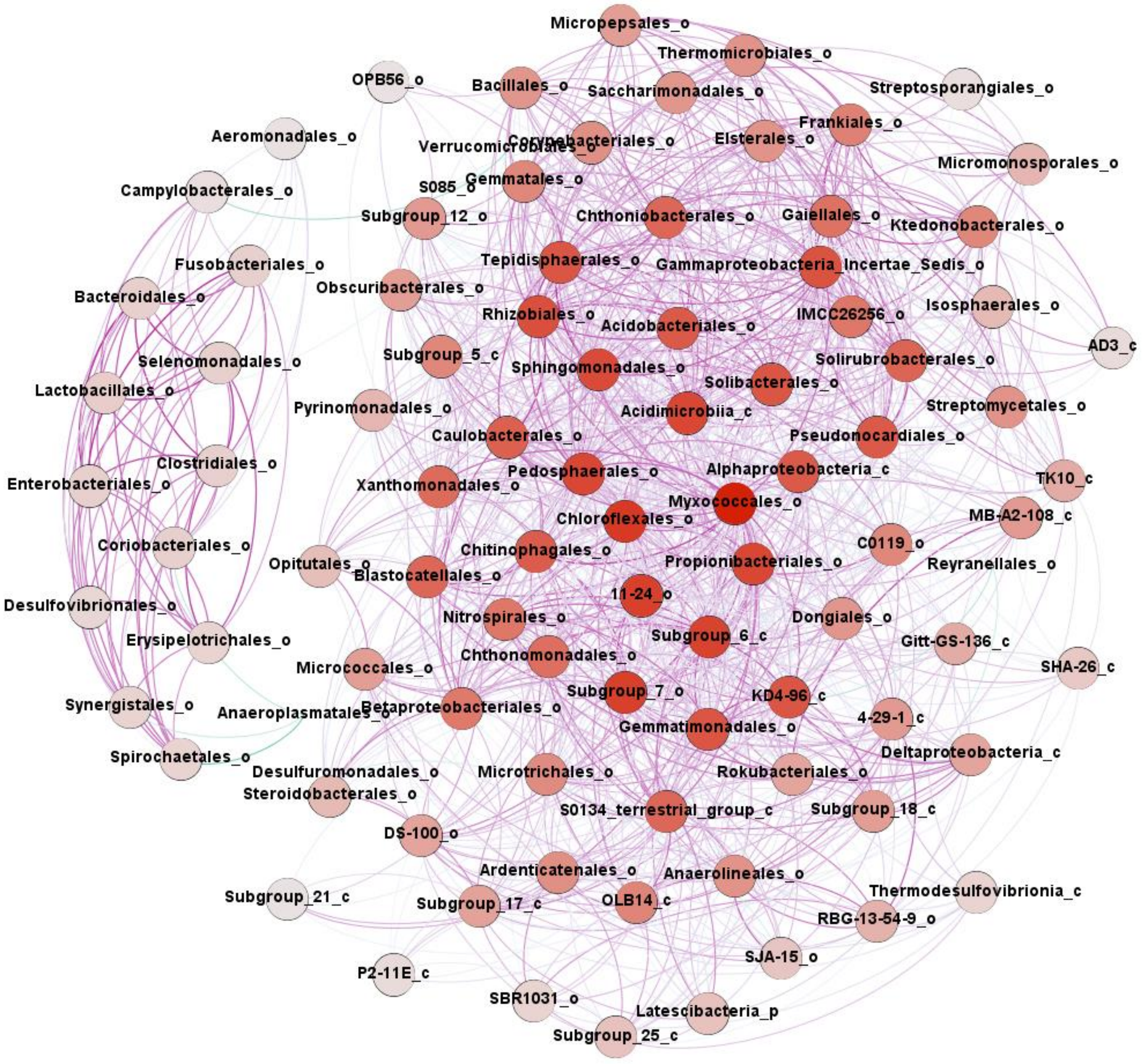

Fig. S10. Bacterial community networks at order level among all samples. Unclassified orders are marked with _c and _p to refer to known class, and phylum, respectively. Co-occurrence nodes and connections were calculated based on Spearman's correlation using $\rho \geq 0.6$ and false discovery rate adjusted *p*-value < 0.01). Thick lines highlight a strong connection.

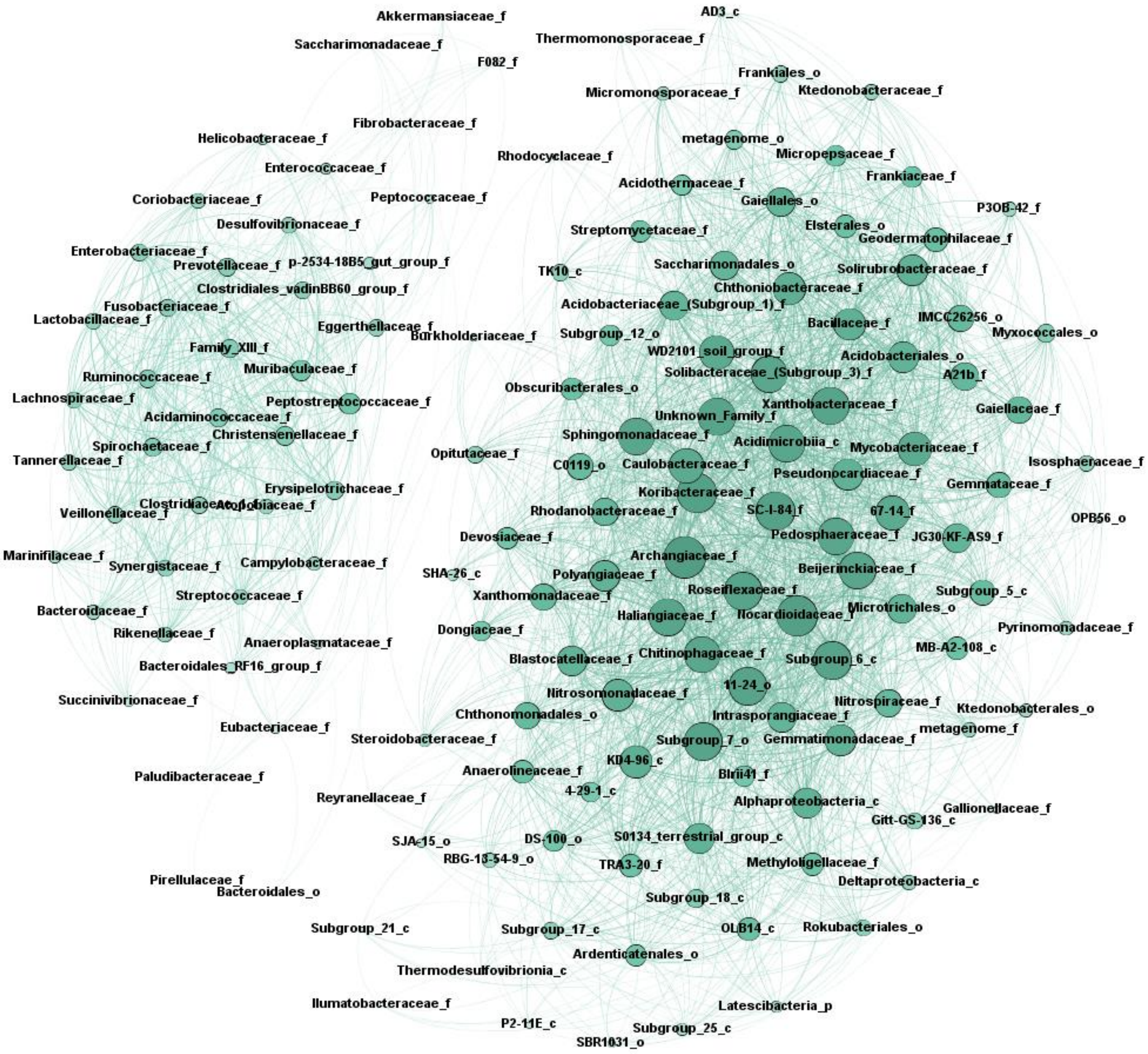

Fig. S11. Bacterial community networks at family level among all samples. Unclassified families were marked with _o, _c, _p to refer to known order, class, and phylum, respectively. Co-occurrence nodes and connections were calculated based on Spearman's correlation using $\rho \geq 0.6$ and false discovery rate adjusted *p*-value < 0.01). Thick lines highlight a strong connection.

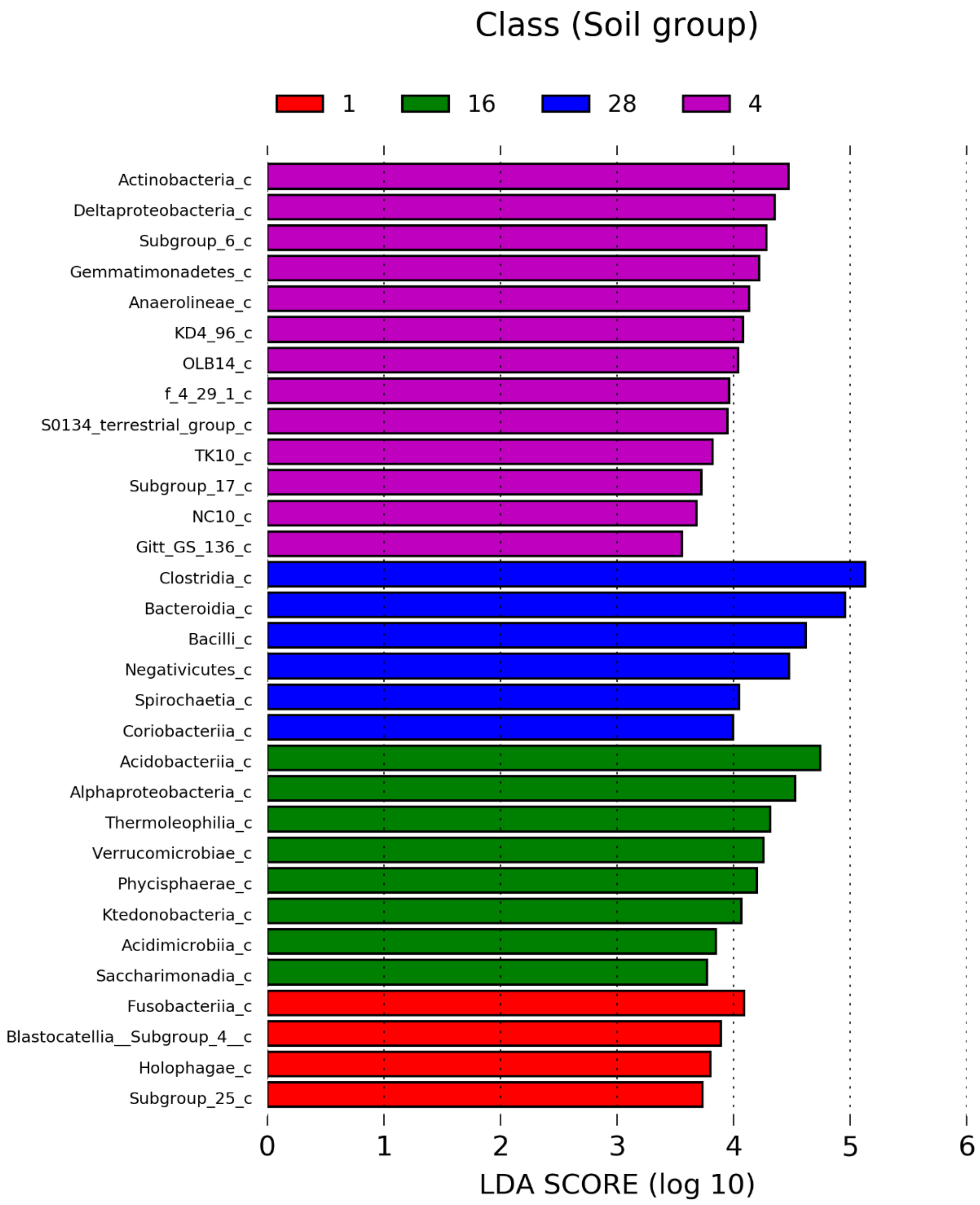

Fig. S12. Differential abundances of bacterial communities at class level (LDA score > 2) based on LEfSe from different soil groups ($n = 30$, $p < 0.05$).

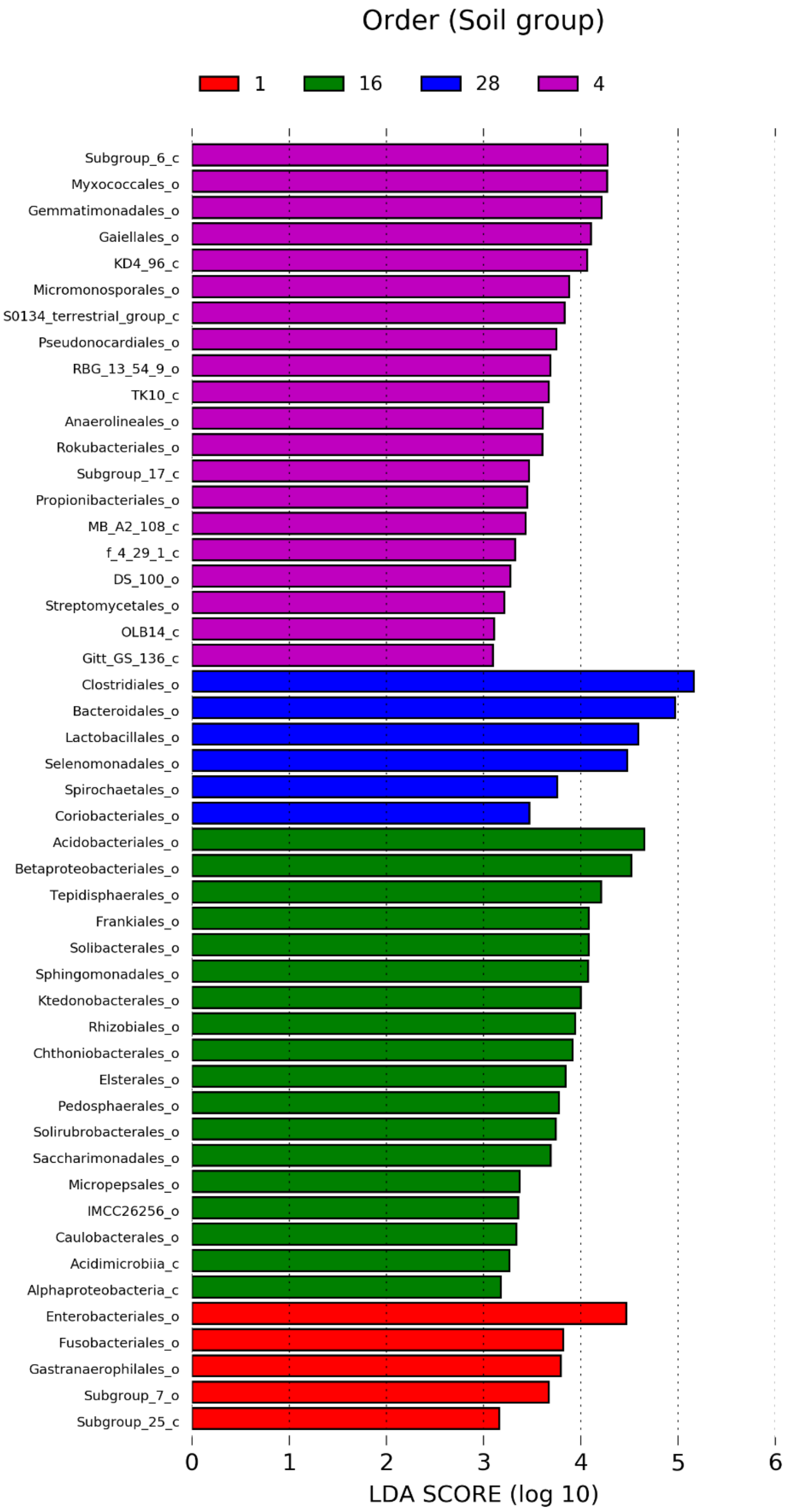

Fig. S13. Differential abundances of bacterial communities at order (LDA score > 2) based on LEfSe from different soil groups (n = 30, p < 0.05).

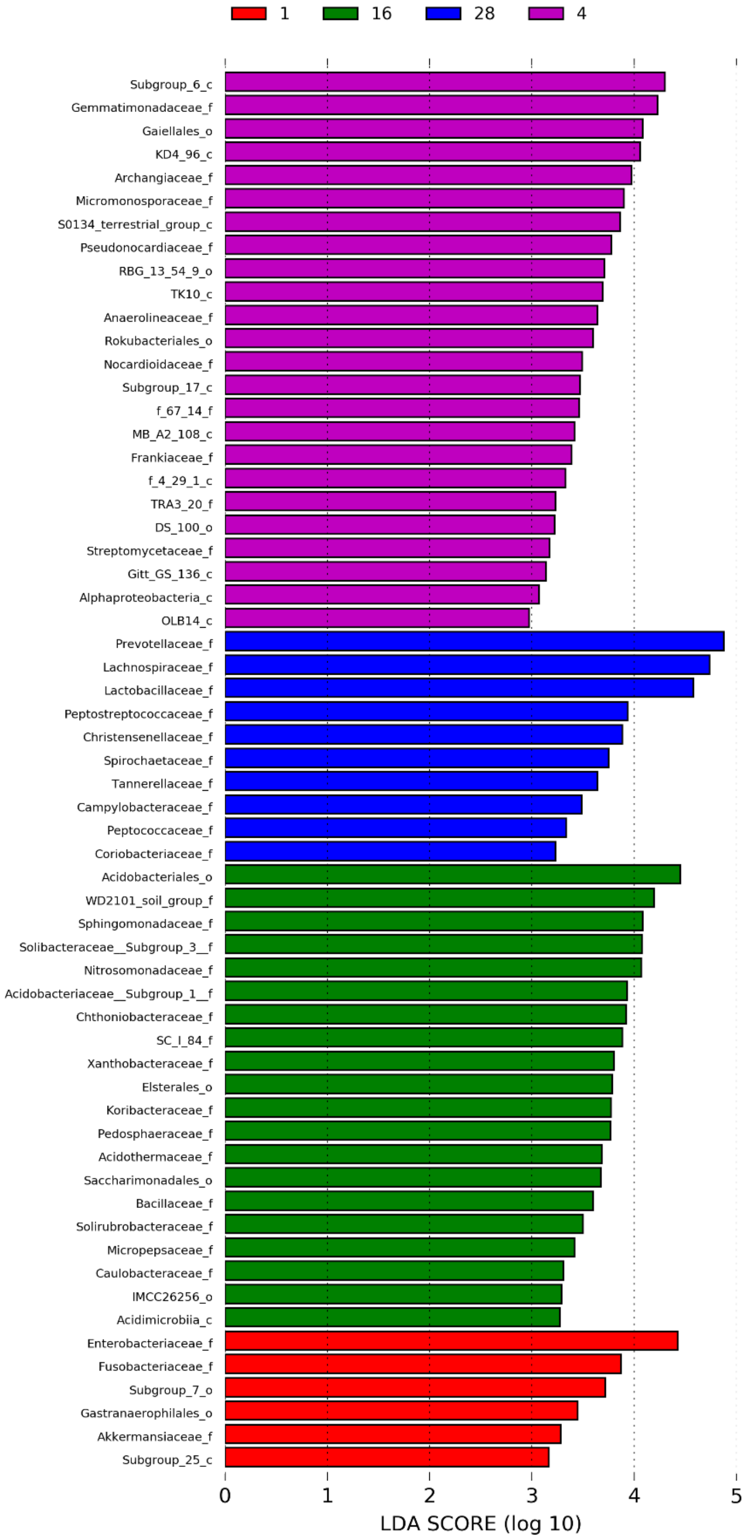

Fig. S14. Differential abundances of bacterial communities at family level (LDA score > 2) based on LEfSe from different soil groups ($n = 30$, $p < 0.05$).
